# Quantitative trait locus mapping combined with variant and transcriptome analyses identifies a cluster of gene candidates underlying the variation in leaf wax between upland and lowland switchgrass ecotypes

**DOI:** 10.1007/s00122-021-03798-y

**Published:** 2021-03-24

**Authors:** Peng Qi, Thomas H. Pendergast, Alex Johnson, Bochra A. Bahri, Soyeon Choi, Ali Missaoui, Katrien M. Devos

**Affiliations:** 1 Department of Plant Biology, University of Georgia, Athens, GA 30602 USA; 2 Institute of Plant Breeding, Genetics and Genomics, and Department of Crop and Soil Sciences, University of Georgia, Athens, GA 30602 USA; 3 Department of Genetics, University of Georgia, Athens, GA 30602 USA; 4 Present Address: Department of Plant Pathology, University of Georgia, Griffin, GA 30223 USA

## Abstract

**Key message:**

Mapping combined with expression and variant analyses in switchgrass, a crop with complex genetics, identified a cluster of candidate genes for leaf wax in a fast-evolving region of chromosome 7K.

**Abstract:**

Switchgrass (*Panicum virgatum* L.) is a promising warm-season candidate energy crop. It occurs in two ecotypes, upland and lowland, which vary in a number of phenotypic traits, including leaf glaucousness. To initiate trait mapping, two F_2_ mapping populations were developed by crossing two different F_1_ sibs derived from a cross between the tetraploid lowland genotype AP13 and the tetraploid upland genotype VS16, and high-density linkage maps were generated. Quantitative trait locus (QTL) analyses of visually scored leaf glaucousness and of hydrophobicity of the abaxial leaf surface measured using a drop shape analyzer identified highly significant colocalizing QTL on chromosome 7K (Chr07K). Using a multipronged approach, we identified a cluster of genes including Pavir.7KG077009, which encodes a Type III polyketide synthase-like protein, and Pavir.7KG013754 and Pavir.7KG030500, two highly similar genes that encode putative acyl-acyl carrier protein (ACP) thioesterases, as strong candidates underlying the QTL. The lack of homoeologs for any of the three genes on Chr07N, the relatively low level of identity with other switchgrass KCS proteins and thioesterases, as well as the organization of the surrounding region suggest that Pavir.7KG077009 and Pavir.7KG013754/Pavir.7KG030500 were duplicated into a fast-evolving chromosome region, which led to their neofunctionalization. Furthermore, sequence analyses showed all three genes to be absent in the two upland compared to the two lowland accessions analyzed. This study provides an example of and practical guide for trait mapping and candidate gene identification in a complex genetic system by combining QTL mapping, transcriptomics and variant analysis.

**Supplementary Information:**

The online version contains supplementary material available at 10.1007/s00122-021-03798-y.

## Introduction

Biofuels, including bioethanol, biodiesel and biogas, present a potential solution to the surge in energy demand caused by population expansion (Yuan et al. 2008). Bioethanol is environmentally friendly and can be generated from a variety of sources such as starch, sugars and cellulose (Bhatia et al. 2017). The first generation bioenergy crops used in the production of bioethanol were mainly food crops, including sorghum, maize, oil crops and sugarcane. However, food crops are an unsustainable source for bioenergy because of the food–fuel conflict and high input requirements. The second generation of herbaceous bioenergy crops are therefore mostly grasses that are adapted to harsher environments, require fewer inputs and, when perennials, increase soil carbon and reduce soil erosion (Oliver et al. 2009).

Switchgrass (*Panicum virgatum* L.) is a warm-season perennial C4 grass native to the USA, and a promising candidate energy crop because of its high biomass production, ability to grow on marginal lands and wide potential cultivation range across the USA. The distribution of switchgrass ranges from latitude 55° N to Mexico and east of the Rocky Mountains (Wullschleger et al. 2010). Switchgrass has traditionally been used as a summer grazing forage crop, for habitat restoration, and for soil and water conservation (Sanderson and Adler 2008; Wright and Turhollow 2010). However, it is only since its emergence as a bioenergy crop that genetic and genomic analyses of this crop have started in earnest.

Switchgrass is an obligate outcrossing species that, based on phenotypic differences and adaptation range, has traditionally been divided into two ecotypes, upland and lowland. Phenotypic characterization of a diverse germplasm collection across a latitudinal gradient recently led to the recognition of a third ‘coastal’ ecotype which had upland leaf characters and lowland architecture (Lovell et al. 2021). Upland ecotypes grow mainly in the northern part of the USA and are usually octoploid (2*n* = 8*x* = 72), although tetraploid (2*n* = 4*x* = 36) upland populations have also been identified (Brunken and Estes 1975). Lowland (and coastal) ecotypes are found at southern latitudes and are predominantly tetraploid (2*n* = 4*x* = 36) (Hultquist et al. 1996; Lewandowski et al. 2003; Lovell et al. 2021). The late flowering lowland ecotypes can capitalize on the longer growing season of the Southern USA leading to higher biomass production, but lack the cold tolerance that ensures survival of the upland ecotypes in the Northern USA (Casler et al. 2007; Wullschleger et al. 2010). Lowland ecotypes also differ from upland accessions by their bluish tint caused by the presence of abaxial leaf wax, the primary components of which are C33 β-diketone (tritriacontane-12,14-dione) and C33 hydroxy-β-diketone (5-hydroxytritriacontane-12,14-dione) (Bragg et al. 2020; Tulloch and Hoffman 1980; Weaver et al. 2018). This wax layer forms a hydrophobic barrier that can protect plants from insect herbivory and pathogen infections. Furthermore, cuticular wax enhances a plant’s drought and heat tolerance by limiting transpiration and reflecting solar radiation (Jenks et al. 1995; Reicosky and Hanover 1978; Riederer and Schreiber 2001). Identifying the genetic factors that are associated with stress tolerance and biomass production is important for breeding switchgrass varieties with high yields and global adaptation.

Quantitative trait locus (QTL) mapping in switchgrass has been conducted for a number of traits including flowering time, biomass yield and biomass composition (Ali et al. 2019; Dong et al. 2015; Lowry et al. 2019; Poudel et al. 2019). Early mapping was conducted in F_1_ populations with simple sequence repeat (SSR), sequence-tagged sites (STS) and diversity arrays technology (DArT) markers, which yielded relatively low-density linkage maps (Li et al. 2014; Liu et al. 2012; Okada et al. 2010; Serba et al. 2013, 2015). More recently, high-density maps have been built with single-nucleotide polymorphism (SNP) markers generated using genotyping-by-sequencing (GBS) (Ali et al. 2019; Fiedler et al. 2018; Grabowski et al. 2014; Lowry et al. 2019; Lu et al. 2013). However, identifying candidate genes underlying QTL is not straightforward, even in high-density maps. Because phenotypes are subject to environmental effects, QTL intervals are typically quite large and encompass a large number of genes making candidate gene identification difficult. Fine-mapping is time-consuming and is further complicated by the obligate outcrossing nature of switchgrass.

In this paper, we combined high-density genetic and trait mapping with gene annotation information, transcriptome analysis and variant mining to identify putative genes controlling wax formation on the abaxial leaf side in lowland switchgrass. The candidate genes, which encode putative acyl-acyl carrier protein (ACP) thioesterases and a Type III polyketide synthase-like protein, are clustered in a highly dynamic region of switchgrass chromosome 7K (Chr07K). This gene cluster was present in the two lowland accessions and absent in the two upland accessions analyzed. This comprehensive study presents the first report of the discovery and rigorous evaluation of a gene cluster underlying a crucial stress tolerance trait in switchgrass, a species with complex genetics, through the integration of multiple datasets.

## Material and methods

### Genetic mapping

#### Mapping populations

Previously, an F_1_ mapping population was generated by crossing the lowland Alamo genotype AP13 with the upland Summer genotype VS16 (Serba et al. 2013). F_1_ progeny PV317 (♀) and PV458 (♂) were crossed to generate an F_2_ population consisting of 475 individuals. We will refer to this population as Pop1. A second F_2_ population (Pop2) was developed by crossing F_1_ progeny PV346 (♀) and PV304 (♂) and consists of 330 progeny. The F_1_ lines used as parents in each of the populations were chosen because they had different levels of colonization by mycorrhizal fungi. Three replications of Pop1 and one replicate of Pop2 were planted in 2015 at the University of Georgia (UGA)’s Iron Horse Farm, Watkinsville, GA. Each replicate was planted in a complete randomized design with 1 m spacing between plants. The following year, Pop2 plants were split to provide material for an additional three replicates. One copy of Pop2 was also maintained in 30-cm-diameter pots in a UGA Plant Biology greenhouse under natural light conditions and approximate temperatures of 27 °C during the day and 21 °C at night.

#### DNA extraction and GBS library preparation

Healthy leaves were collected from the field-grown parents and F_2_ progeny, flash-frozen in liquid nitrogen and stored at − 80 °C until further use. The leaves were ground to a fine powder using a TissueLyserII bead mill (Qiagen) and genomic DNA was isolated using a CTAB procedure. DNA quantity and quality were determined by Nanodrop spectrophotometer and agarose gel electrophoresis, respectively. DNA samples were diluted in 1/10th TE buffer to a concentration of 50 ng/µL.

DNA was digested with the restriction enzymes *Pst*I, *Msp*I and *Ape*KI, and barcoded libraries were constructed for each sample as described in Qi et al. (2018). Please note that we no longer recommend the use of a third restriction enzyme to reduce the fragment pool (Qi et al. 2018). Each library was quantified on a Qubit 2.0 fluorometer using the dsDNA High Sensitivity Assay Kit. The quality of each library was verified by agarose gel electrophoresis. Equal amounts (30 ng/sample) of 200 quality-verified samples with a concentration of at least 5 ng/µL were pooled. DNA fragments smaller than 300 bp, including primer dimers, were removed from the pooled libraries using Sera-Mag SpeedBeads (Qi et al. 2018). The pooled libraries were sequenced on an Illumina NextSeq platform (paired-end 150 bp) to yield approximately 400 Mb of sequence per sample.

#### SNP calling and filtering

Read processing, SNP calling and filtering were essentially done as described in Qi et al. (2018). Briefly, the sequence reads were split by barcode, trimmed to remove adapters, barcodes and low-quality bases, and aligned to the switchgrass AP13 reference genome v2.0 (pre-release access provided by the Joint Genome Institute) with Bowtie2 using the parameters*—maxin 900—no-discordant—no-mixed*. SNP calling was done using the UnifiedGenotyper module in the Genome Analysis Toolkit (GATK) version 3.4 (McKenna et al. 2010) using *dcov 1000-glm BOTH*. Because we used both single-nucleotide polymorphisms (SNPs) and insertions/deletions (InDels) in our study, we will refer to variants globally as nucleotide polymorphisms (NPs). NP filtering included the removal of NPs with three or more alleles, a quality depth (QD) value less than 10, and allele frequencies lower than 10% or higher than 90%. Read counts for the filtered NPs were converted to the mapping scores A (homozygous parent 1), B (homozygous parent 2), H (heterozygous), D (A or H) and C (B or H) using the script SNP_Genotyper.py (Qi et al. 2018). NPs with a read depth of less than 8X were treated as missing data. The script SNP_Genotyper.py also consolidated NPs that were located within 1,000 bp into a single representative marker (Qi et al. 2018). After the removal of NPs with missing data in more than 30% of the samples, NPs were tested for cosegregation using the script SNP_cosegegration.py (Qi et al. 2018)*.* Within a group of cosegregating markers, the NP with the least number of missing or ambiguous (C and D) genotypic scores was retained for generation of the framework map.

#### Creating maternal (HA), paternal (AH) and HH nucleotide polymorphism datasets

Chi-square tests were used to determine the markers’ deviation from expected Mendelian segregation ratios. Highly distorted markers (*p*-value ≤ 1*e*^−10^) were discarded. The remaining NPs were categorized according to their segregation ratio and parental genotype. NPs that were heterozygous in both parents with progeny segregating A:H:B (1:2:1) comprised the HH dataset. NPs with genotypic score A or B in parent 1 and H in parent 2, and segregating A:H or B:H (1:1) were combined, and B scores were converted to A scores to form the paternal (AH) dataset. Similarly, H:A and H:B markers were combined, and B scores were converted to A scores to yield the maternal (HA) dataset.

#### Genetic map construction

The maternal, paternal and HH genetic maps were constructed using a combination of MSTMap (Wu et al. 2008) and MAPMAKER (modified from Lander et al. 1987) as described by Qi et al. (2018). The population type was set as ‘backcross’ for the maternal and paternal datasets, and as ‘F2 intercross’ for the HH dataset. Cosegregating markers were added to the framework map to the same location as their representative marker using an in-house python script. Linkage maps were drawn with MapChart (Voorrips 2002).

#### Linkage map-based comparative analyses

Regions spanning 500 bp on either side of mapped NPs were extracted from the switchgrass v2.0 genome sequence and used as queries in BLASTN searches against the genome assemblies of *Panicum virgatum* v4.0 and v5.1, *Sorghum bicolor* v3.1.1, *Oryza sativa* v7.0, *Setaria italica* v2.2 and *Brachypodium distachyon* v3.0. All assemblies were downloaded from Phytozome (phytozome-next.jgi.doe.gov/). For each query, the best hit in each assembly with an *e*-value ≤ 1*e*^−5^ was recorded and used to generate comparative dot plots.

### QTL mapping

#### Leaf wax and hydrophobicity phenotyping

Each plant from each of three replicates of Pop2 was assessed visually in 2018 in the field at the R1 developmental stage (Moore et al. 1991) for abaxial leaf waxiness using a scale from 1 to 4, where 1 refers to a leaf with a glossy green color (low wax level) and 4 refers to a leaf with a dull bluish color (high wax level). In addition, contact angles of water droplets, which indicate leaf hydrophobicity, were used as a proxy for measuring leaf waxiness of F_2_ progeny of Pop2 maintained in the greenhouse. A test was first performed on both grandparents of Pop2 (AP13 and VS16) and on nine F_2_ progeny by measuring the left and right contact angles of 5 µL water droplets on both the adaxial and the abaxial leaf surfaces using a Kruss DSA100 Drop Shape Analyzer. The measurements were taken on a single healthy and freshly harvested leaf from internodes 3–5 at 5, 6, 7, 8 and 9 cm from the base of the leaf blade. Data from the adaxial and abaxial leaf surfaces were analyzed with a two-way ANOVA using the lmer4 package in R version 4.0.3 (R Development Core Team 2012) to check for the effects of ‘genotype’ and ‘leaf side’ (adaxial *vs.* abaxial) on the droplet contact angle. The left and right droplet contact angles were averaged, and position of measurement was nested within the sample as a random effect. In addition, at each position, the Pearson’s correlation coefficient of the droplet contact angles between the adaxial and the abaxial leaf surfaces was calculated and the corresponding *p*-values of the correlations were generated using the rcorr function in the Hmisc package in R version 3.2.2 (R Development Core Team 2012). Leaf hydrophobicity measurements were then extended for the abaxial leaf surface at 5, 6, 7, 8 and 9 cm from the base of the leaf blade to both parents (PV304 and PV346) and a subset of 145 of the 330 F_2_ progeny that were maintained in the greenhouse for QTL analysis.

#### QTL mapping

The visual wax scores and droplet contact angle values measured on the abaxial leaf side of the progeny of Pop2 were used as inputs for QTL analysis. QTL mapping was conducted with Windows QTL Cartographer 2.5 (Wang et al. 2012a) using the composite interval mapping model. The population type for QTL analysis in the HH maps was set as ‘SF2,’ and as ‘B2’ for the maternal and paternal maps. A walk speed of 0.5 cM was used for QTL identification. The LOD threshold for significant QTL (*p* ≤ 0.05) was determined by 1000 permutations.

### Expression analyses

#### RNASeq and differential expression analysis

Eight plants each of two tetraploid lowland accessions (Alamo and Kanlow) and two tetraploid upland accessions (Summer and Dacotah), were grown from seed (purchased from Applewood Seed Company, Arvada, CO) in a controlled environment chamber under 17-h days (25 °C) and 7-h nights (22 °C) for 12 weeks. Plants were then subjected to 16-h long days for one week. The most recently emerged leaf from each plant was harvested one hour after the light was switched on, and four leaves per genotype were pooled to constitute one replicate for a total of two replicates per accession.

Total RNA was extracted from the pooled leaves using Trizol. Remnant genomic DNA was removed with the Turbo DNA-free kit (Invitrogen) according to the manufacturer’s instructions. Barcoded libraries for RNASeq were generated with the KAPA Stranded mRNASeq kit (Kapa Biosystems) using 1.5 µg of input RNA. Quality control of the libraries was done on a Fragment AnalyzerTM Automated CE System and by qPCR. Uniquely barcoded libraries were pooled and sequenced on an Illumina NextSeq platform (75 bp paired-end reads) at the Georgia Genomics and Bioinformatics Core.

The raw sequencing reads were split by barcode, trimmed to remove adapter sequences and low-quality sequences (Phred score < 33) using the paired-end mode of Trim Galore (version 0.45, https://github.com/FelixKrueger/TrimGalore). The trimmed reads were aligned against the switchgrass AP13 reference genome v5.1 (https://phytozome-next.jgi.doe.gov/) with HISAT2 (version 2.1.0; Kim et al. 2015) using the parameters*—phred33—max-intronlen 50000—novel-splicesite*. Differential expression analyses were done by comparing transcript levels in the two lowland accessions *versus* the two upland accessions using DESeq2 (Love et al. 2014). Briefly, the two Alamo replicates and two Kanlow replicates were treated as four lowland replicates. Similarly, the two Summer replicates and two Dacotah replicates were treated as four upland replicates. The aligned reads were assembled into transcripts with the guidance of the switchgrass v5.1 annotation (Lovell et al. 2021) using StringTie (Pertea et al. 2015). Transcripts were merged across samples and used as reference for transcript quantification. Quantified GTF files were converted to a gene count matrix (Pertea et al. 2016), which was then used for differential expression (DE) analyses with DESeq2.

#### Validation by qRT-PCR of genes differentially expressed between ecotypes

Quantitative real-time PCR (qRT-PCR) was used to validate differential expression between ecotypes observed from the RNASeq data using RNA extracted from ~ 13-week-old plants of the same accessions, grown from seed under the same conditions as the plants for RNASeq, except that they were placed under short days (13-h days) for one week prior to harvest, and samples were harvested one hour after the start of the dark period. First-strand cDNA synthesis was primed with anchored oligo(dT) primers (Invitrogen) and conducted using RevertAid Reverse Transcriptase (Thermo Scientific™) according to the manufacturer’s instructions. Primers specific to Pavir.7KG077009 (CER_7K_F: 5′-TGCATTGAAGGCCAACTTCA-3′ and CER_7K_R: 5′-TGGTATTGCTTGACCCGTCC-3′) were designed against regions that were conserved between upland and lowland accessions and to be subgenome specific. The reference gene used for qRT-PCR, Pavir.5KG045800, was selected from the RNASeq dataset because it had a similar expression level across all the samples (adjusted *p*-values > 0.5) (Primers Ref2_5K_MSTRG.32677.1_F: 5′-CTGAACCCAACTCCATCAAC-3′ and Ref2_5K_MSTRG.32677.1_R: 5′-GTGCACTTGCTCCTGTCAGC-3′). qRT-PCR reactions were performed in a 20 µL volume consisting of 1X SsoAdvanced™ Universal Inhibitor-Tolerant SYBR Green Supermix (Bio-Rad), 500 nM each of forward and reverse primer, and 30 ng of cDNA template. The qRT-PCR conditions were initial denaturation at 98 °C for 3 min followed by 40 cycles of denaturation at 98 °C for 10 s, and annealing and extension at 60 °C for 30 s. A melting curve analysis was performed upon completion of the PCR to test for the presence of non-specific amplification products.

### Gene duplication and nucleotide variant analyses

#### Nucleotide polymorphism calling and annotation of AP13 and VS16 resequencing data

To identify coding sequence variants between the grandparents of the mapping population, resequencing reads of AP13 (SRR8440701, 92 million reads, paired-end 150 bp) and VS16 (SRR3947377, 197 million reads, paired-end 150 bp) were downloaded from NCBI SRA and aligned to the switchgrass AP13 genome assembly v5.1 using Bowtie2 (Langmead and Salzberg 2012). The Bowtie2 parameters were set as ‘*—maxin 900—no-discordant—no-mixed’*. Variant calling was done with GATK (McKenna et al. 2010) as described for the GBS data. Nucleotide variants with three or more alleles and NPs with a quality depth (QD) ≤ 10 were removed. The remaining NPs were annotated using SNPEff (Cingolani et al. 2012) based on the switchgrass v5.1 annotations (Lovell et al. 2021) to predict the effect of each variant.

### Nucleotide polymorphisms and presence/absence

Raw Chr07K NPs identified in a set of 95 lowland and 65 upland accessions (see Supplementary Table S1 for the germplasm list) were provided by Lovell and colleagues (Lovell et al. 2021) and filtered to remove NPs missing in more than 50% of the accessions. To obtain a measure of the level of variation present in the coding region of our target genes across lowland accessions, we annotated NPs that were located in the genes of interest, and had a minor allele frequency ≥ 0.05 and ≤ 30% missing data in the set of lowland germplasm using SNPEff (Cingolani et al. 2012). We also calculated the NP absence ratio in both the upland and lowland sets of accessions. For each NP site, we calculated and plotted the percentage of NPs that were missing in ≥ 50% of accessions within an ecotype per 10 Kb region.

#### Gene duplication analysis

To determine whether the genes present in the wax region were more likely to have arisen through duplication, we examined whether other gene copies were present in the switchgrass genome. The protein sequences corresponding to the genes annotated on Chr07K were retrieved from Phytozome (https://phytozome-next.jgi.doe.gov/) and used as queries in BLASTP searches against the primary proteins from Chr07N. Top hits with an *e*-value ≤ 1*e*^−5^ were retained. Chr07N-Chr07K protein pairs were used for syntenic block identification using the software MCScanX (Wang et al. 2012b) with a match score of 50, match size of 3, gap penalty of − 1, overlap window of 5, *e*-value threshold of 1*e*^−5^ and maximum gap of 25 as parameters. Protein pairs located in a syntenic block were considered homoeologs.

Chr07K proteins were also used as queries in a BLASTP analysis against all annotated proteins in the switchgrass v5.1 assembly. The top hit (excluding self hits) with an *e*-value ≤ 1*e*^−5^, an alignment length ≥ 70% of the query length, ≥ 80% similarity with the query sequence and not located in a Chr07N-Chr07K synteny block were considered paralogs.

The percentage of Chr07K genes with a homoeolog on Chr07N and the percentage of genes with a paralog in the switchgrass genome (homoeologs were excluded) were calculated per 200 Kb and plotted.

## Results

### Nucleotide polymorphims for genetic map construction

A total of 1,807,974 SNPs and InDels were obtained from GBS data for Pop1, and 1,858,673 SNPs and InDels for Pop2. The GBS variants identified between the grandparents AP13 and VS16 were distributed across the AP13 reference genome and corresponded largely to the gene density (Supplementary Figure S1). Both SNPs and InDels [globally referred to as nucleotide polymorphisms (NPs)] were used for mapping. After filtering and consolidation, a total of 16,591 markers remained for Pop1 and 13,098 markers for Pop2. These NP markers were classified into three subgroups, heterozygous in both parents (10,862 HH markers for Pop1 and 8996 for Pop2; their distribution across the genome is shown in Supplementary Figure S1), paternal heterozygous (2976 AH markers for Pop1 and 1913 for Pop2) and maternal heterozygous (2753 HA markers for Pop1 and 2189 for Pop2). Each of the three marker subgroups was used for linkage map construction.

### Switchgrass genetic maps

**Fig. 1:**
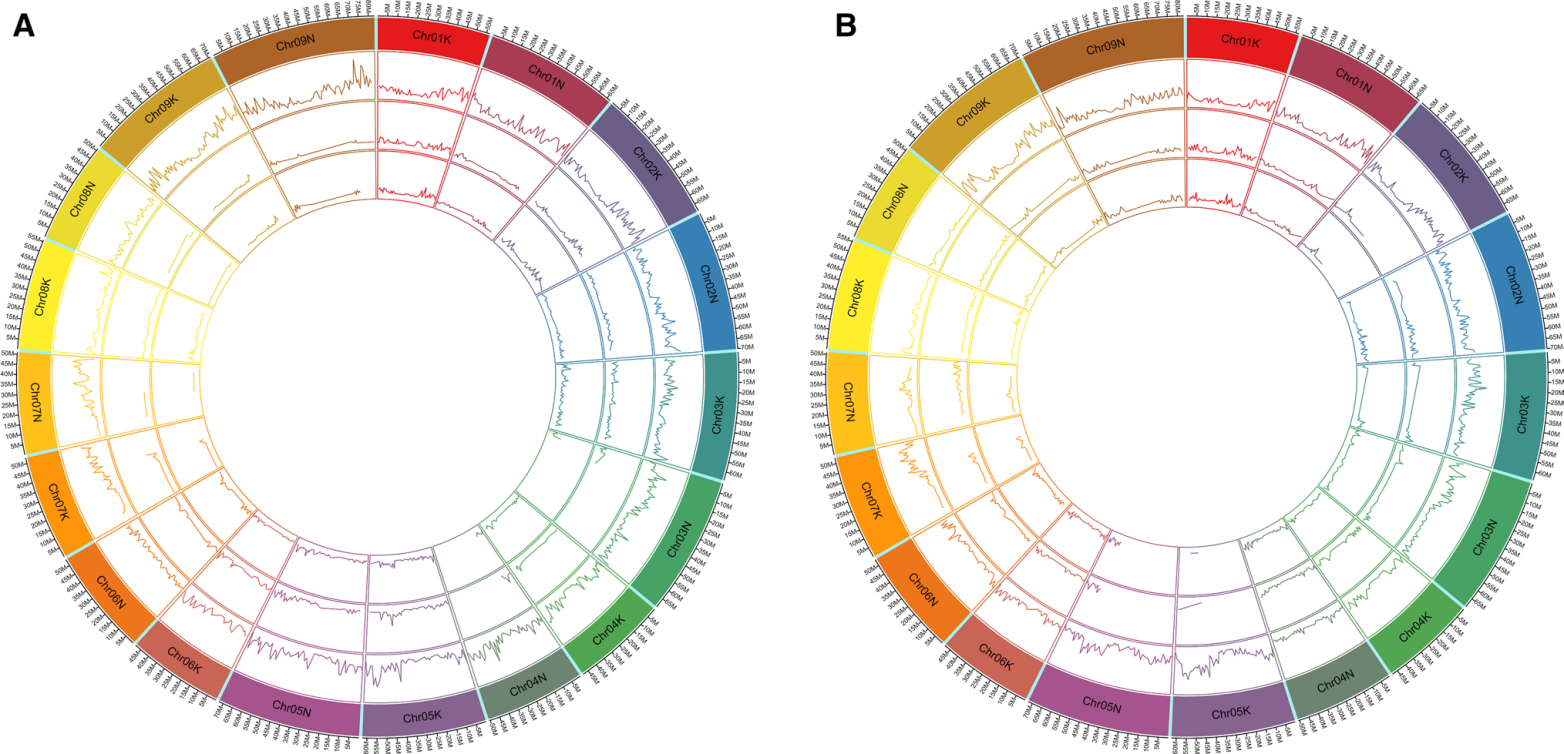
Distribution of markers (per Mb, height min = 0, height max = 30) in, from outer ring to inner ring, the HH, maternal (HA) and paternal (AH) maps generated in Pop1 (**a**) and Pop2 (**b**) relative to the switchgrass AP13 reference genome (colored blocks) (color figure online)

The HH maps for Pop1 and Pop2 covered 1382 cM and 1454 cM, respectively, and each consisted of 18 linkage groups corresponding to the 18 chromosomes of switchgrass (Table 1; Fig. 1; Supplementary Figures S2 and S3). The majority of the AH and HA linkage groups (Supplementary Figures S4–S7), however, were incomplete to varying degrees (Fig. 1; Supplementary Figure S8) due to a lack of markers that were homozygous in one parent and heterozygous in the other. The missing regions varied between the two populations except for the intervals 35.7 Mb—chromosome end on Chr07K and 25.3–55.5 Mb on Chr09N which were absent in both the AH and HA maps in Pop1 as well as Pop2 (Fig. 1; Supplementary Figure S8).

**Table 1:** Marker number and length of HH, paternal and maternal maps for Pop1 and Pop2

Switchgrass chromosome	Pop1						Pop2					
	HH map		Paternal (AH) map		Maternal (HA) map		HH map		Paternal (AH) map		Maternal (HA) map	
	Number of markers	Map length (cM)	Number of markers	Map length (cM)	Number of markers	Map length (cM)	Number of markers	Map length (cM)	Number of markers	Map length (cM)	Number of markers	Map length (cM)
Chr01K	270	74.8	144	67.9	144	78.2	179	80.9	147	70.8	131	86.2
Chr01N	377	74.2	58	51.4	55	59.1	329	84.1	80	80.0	75	88.0
Chr02K	355	81.5	109	52.5	124	93.8	387	110.7	40	36.7	33	47.0
Chr02N	410	82.3	67	69.8	59	90.5	314	90.0	103	73.4	114	73.0
Chr03K	321	87.1	139	76.0	124	95.5	399	104.7	38 + 14	26.4 + 9.9	13 + 36	8 + 36.4
Chr03N	447	88.0	20	14.3	21	15.5	357	88.5	50	73.2	65	92.2
Chr04K	240	76.3	34	52.1	47	61.8	165	58.5	74	52.8	55	49.5
Chr04N	361	58.7	28	17.1	20	18.0	122	57.6	100	59.4	94	67.0
Chr05K	332	99.9	138	85.5	135	112.3	552	99.3	12	2.7	0	0.0
Chr05N	420	93.6	126	79.9	94	95.6	424	102.8	42	28.4	48	36.5
Chr06K	213	61.0	76	69.0	48	71.8	190	63.1	59	56.4	88	49.1
Chr06N	166	50.2	59	50.6	57	53.7	163	59.8	50	48.0	57	49.6
Chr07K	297	65.2	25	18.2	13	17.0	285	55.5	17	15.4	15	9.3
Chr07N	296	66.1	10	7.6	0	0.0	81	44.3	103	54.0	27	5.4
Chr08K	114	51.8	50	53.0	56	44.8	124	74.3	40	48.3	41	43.8
Chr08N	169	65.3	9	6.7	7	9.3	69	51.1	63	53.9	61	55.5
Chr09K	619	99.5	36	22.4	37	35.6	565	97.7	60	82.2	70	107.9
Chr09N	621	106.3	57	101.3	47	95.2	449	130.9	100	97.4	112	103.9
Total	6028	1381.8	1185	895.3	1088	1047.7	5154	1453.8	1192	969.3	1135	1008.3

It should be noted that only one marker per group of cosegregating markers is included in the linkage maps presented in Supplementary Figures S2 to S7 for ease of readability. The full maps, including cosegregating markers together with the genotypic scores of the NP variants, are given in Supplementary Table S2. The NP variants together with 500 bp of flanking sequence are given in Supplementary Table S3.

### Comparative relationships between the switchgrass linkage maps, and switchgrass and other grass genome assemblies

**Fig. 2:**
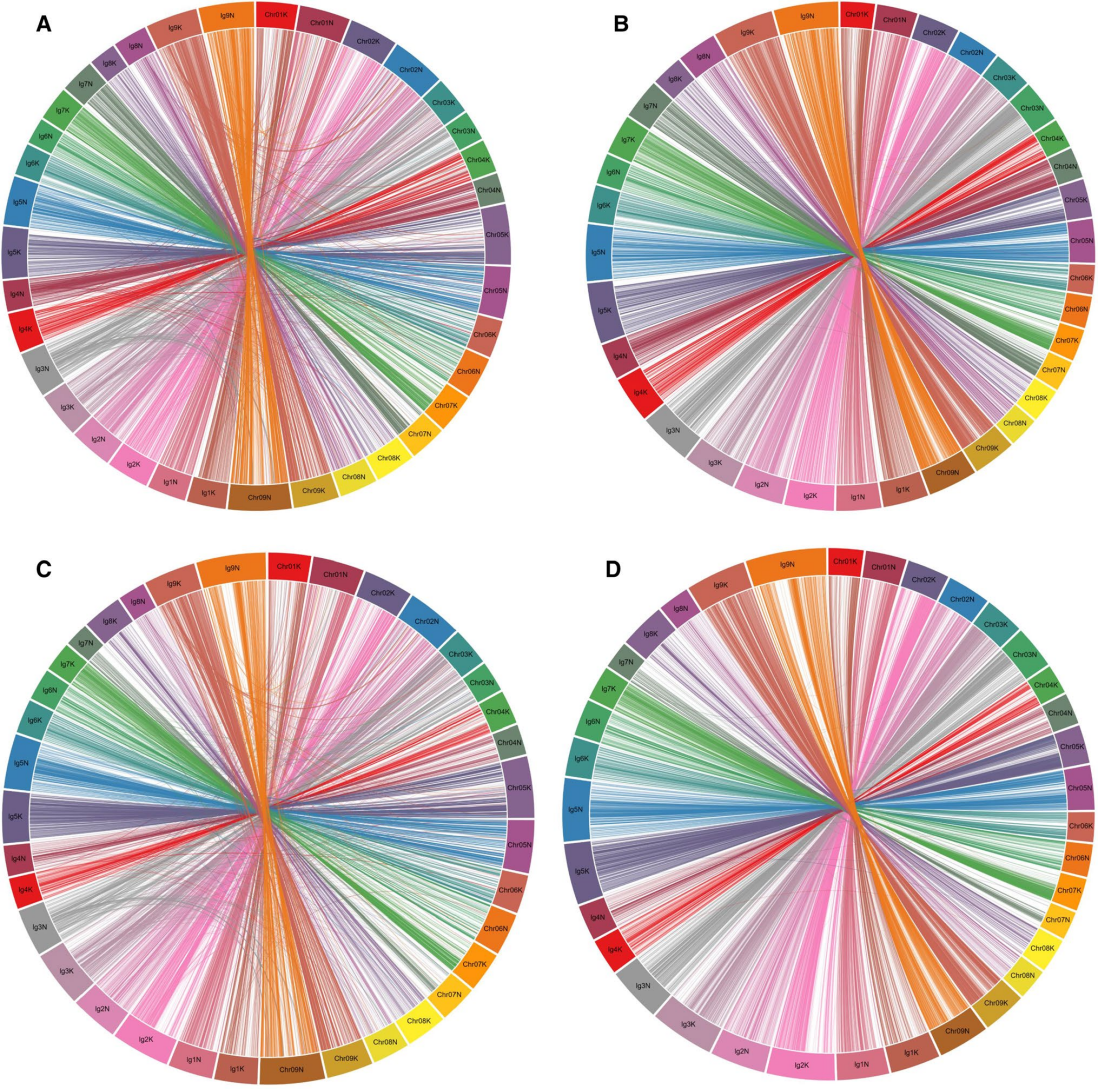
Circos diagrams showing the relationship of switchgrass AP13 genome assembly v4.0 (**a, c**) and assembly v5.1 (**b, d**) with the HH linkage map generated in Pop1 (**a, b**) and the HH linkage map generated in Pop2 (**c, d**)

The NPs used in this study were initially called against the switchgrass v2.0 genome sequence and later mapped to v4.0 and v5.1 by BLASTN analysis (Supplementary Table S2), allowing us to assess the level of improvement between subsequent versions of the switchgrass AP13 genome assembly. Discrepancies between the genetic maps and assembly v2.0 were observed for 31.4% markers in the HH Pop1 map and 29.1% markers in the HH Pop2 map. These numbers dropped to 28.1% and 27.9% in Pop1 and Pop2, respectively, for assembly v4.0, and to 1.0% and 1.3% for v5.1, indicating the high quality of assembly v5.1 (Fig. 2). All markers from the six switchgrass linkage maps were also mapped to the genome assemblies of *Sorghum bicolor* v3.1.1, *Oryza sativa* v7.0, *Setaria italica* v2.2, and *Brachypodium distachyon* v3.0 to allow for comparative trait analyses (Supplementary Table S2). The relationships between the switchgrass genetic maps and genome assemblies of selected grass species are depicted in Supplementary Figure S9.

### Nucleotide polymorphisms and differentially expressed genes between AP13 and VS16

We identified 7,298,242 SNPs and 709,521 InDels between AP13 and VS16 from the resequencing data. Their distribution across the genome is shown in Supplementary Figure S10. Of these NPs, 3.2%, 3.2% and 10.9% were located in exons, untranslated regions (UTRs) and introns, respectively. A detailed breakdown of the number of NPs by location and type is given in Supplementary Table S4. Among the polymorphisms identified in the coding region between AP13 and VS16, 298,386 were silent, 430,155 were missense events, and 6095 were nonsense mutations according to the NP annotation by SNPEff (Cingolani et al. 2012). Variants in 11,406 genes were predicted to have a high impact on gene function, and variants in 47,565 genes to have a moderate impact. A total of 8413 genes were shared between the two categories as they carried both high-impact and moderate-impact NPs.

RNASeq analysis identified 7653 genes with significantly (*p* adjusted value < 0.01) different expression levels (> twofold change) in the two lowland accessions (Alamo and Kanlow) compared to the two upland accessions (Summer and Dacotah) under our growth conditions. Of the differentially expressed (DE) genes, 4205 were upregulated in the lowland accessions and 3448 were upregulated in the upland accessions.

The NP data, along with their effect annotation data and differential expression data, were combined into an in-house database. The database was then mined to identify candidate genes for the chromosome 7K leaf wax QTL that was mapped in the AP13 × VS16 mapping population.

### Phenotypic variation for leaf wax and hydrophobicity between AP13 and VS16

**Fig. 3:**
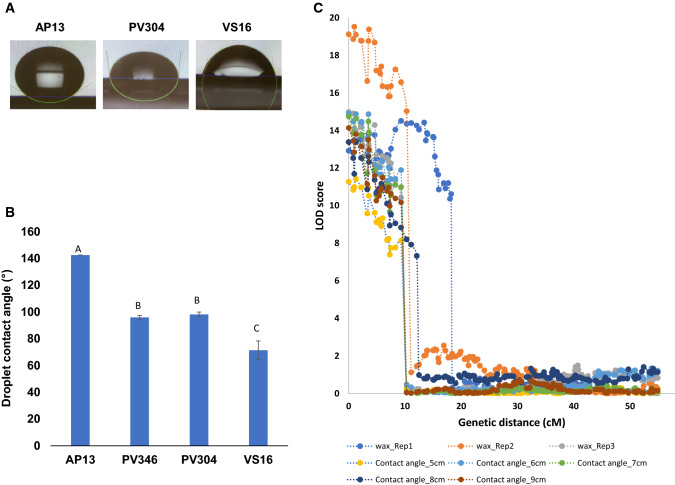
**a** Drop shape image at 5 cm from the base of the leaf blade on the abaxial leaf surface of AP13, VS16, and PV304; **b** Drop contact angle measurements at 5 cm from the base of the leaf blade on the abaxial leaf surface of AP13, VS16, PV304 and PV346; **c** QTL identified on Chromosome 7K for drop contact angles on the abaxial leaf surface and for visual scores of abaxial leaf waxiness

Statistical analyses of the preliminary droplet contact angle measurements that were taken on both grandparents (AP13 and VS16) and nine randomly selected progeny of the F_2_ mapping population showed significant ‘genotype’ (*p* < 2*e*^−16^) and ‘genotype × leaf side’ (*p* < 2*e*^−16^) effects (Supplementary Table S5), indicating that leaf glaucousness in switchgrass is strongly affected by the genotype of the grandparents/F_2_ progeny. Adaxial values varied little between the grandparents (136.5° for AP13 and 132.0° for VS16) and were also relatively consistent across the nine F_2_ genotypes (min 123.2°, max 144.0°). Abaxial contact angles were, on average, 180% higher (*p* = 7.01*e*^−10^) for AP13 (137.7°) than VS16 (51.8°) and had a more than threefold increase in variation in the F_2_ progeny (min 65.9°, max 129.2°) (Fig. 3a; Supplementary Table S6). The F_1_ sibs used as parents, PV346 and PV304, had drop contact angles intermediate between those of the grandparents, AP13 and VS16 (Fig. 3b). No correlation was observed between the droplet contact angles on the abaxial and those on the adaxial leaf surfaces at any distance from the base of the leaf blade [*r*^2^ range: 0.42 (*p* = 0.051) to 0.03 (*p* = 0.8927); Supplementary Table S7]. The droplet contact angle measurements taken for Pop2 at different distances from the leaf base on the abaxial leaf side and averaged across the left and right angles are given in Supplementary Table S8. Visual scores for abaxial leaf waxiness are also included in Supplementary Table S8.

### QTL mapping of leaf wax

Colocalizing QTL for the visual wax scores and hydrophobicity measurements were identified on chromosome 7K (Chr07K) in the region 0 to 10 cM (Fig. 3c). The Chr07K QTL explained between 16 and 35% of the phenotypic variation (average of 19.8% for visual wax scoring and 32.5% for droplet contact angle measurements; Table 2) with the AP13 allele contributing an increase in both traits (Supplementary Figures S11A and S11B). Although very few markers were available in the region 0–20 Mb in all our maps (Fig. 1), the mapping data indicated that recombination was very low in this region. Projection of the marker data onto the switchgrass genome assembly v5.1 showed that the 0–10 cM QTL interval corresponded to the region 0–31 Mb on Chr07K.

**Table 2:** QTL for visually scored leaf waxiness and for the contact angle of water droplets on the abaxial leaf surface

Trait	Chromosome	Left marker	Left marker position (cM)	Right marker	Right marker position (cM)	Marker at highest LOD value	Marker position at highest LOD value (cM)	LOD score	LOD significance threshold	Additive effect	Dominant effect	*R*^2^ (%) at highest LOD value
Visual wax_Rep1	Chr07K	Tag_7893	0	Tag_8222	18.2	Tag_8032	13.4	14.4	4.0	0.239	− 0.016	15.6
Visual wax_Rep2	Chr07K	Tag_7893	0	Tag_8013	10.3	Tag_7893	0	19.1	4.1	0.474	− 0.065	23.6
Visual wax_Rep3	Chr07K	Tag_7893	0	Tag_7941	8.3	Tag_7893	0	15.0	3.9	0.349	− 0.040	20.3
Visual wax_Rep1	Chr09K	Tag_9919	17.9	Tag_12092	18	Tag_12092	18	4.3	4.0	0.093	0.054	4.8
Contact angle_5cm	Chr06N	Tag_7555	40.5	Tag_7555	40.5	Tag_7555	40.5	4.2	4.0	7.261	7.389	8.9
Contact angle_5cm	Chr07K	Tag_7893	0	Tag_7941	8.3	Tag_7893	0	11.2	4.0	21.245	0.407	26.9
Contact angle_6cm	Chr07K	Tag_7893	0	Tag_7941	8.3	Tag_7893	0	14.9	4.2	24.065	− 2.026	34.1
Contact angle_7cm	Chr07K	Tag_7893	0	Tag_7941	8.3	Tag_7893	0	14.7	4.2	23.464	− 4.481	35.2
Contact angle_8cm	Chr07K	Tag_7893	0	Tag_8023	11.1	Tag_7893	0	13.4	4.1	23.552	− 8.244	31.9
Contact angle_9cm	Chr07K	Tag_7893	0	Tag_7941	8.3	Tag_7893	0	14.1	4.2	23.921	− 8.380	34.4
Contact angle_6cm	Chr09K	Tag_11638	89.7	Tag_11633	90.5	Tag_11633	90.5	5.8	4.2	− 14.769	16.224	10.7
Contact angle_9cm	Chr05K	Tag_6067	0	Tag_6002	10	Tag_6067	0	4.4	4.2	13.703	− 8.595	9.2

Unique smaller effect QTL were identified on chromosomes 5K, 6N and 9K (Table 2). These QTL each explained between 5 and 11% of the phenotypic variation, but as they were not consistently identified across replicates or measurements, only the large effect QTL interval on Chr07K was screened for candidate genes.

### Candidate gene prediction for leaf wax QTL

Although the genomic region spanned by the wax QTL was large (31 Mb), the gene density in that region was relatively low with 1377 genes compared to, on average, 2280 genes (range 1696–3339) in the first 31 Mb of the other K genome chromosomes (Supplementary Figure S1). We hypothesized that the gene controlling leaf glaucousness, a trait that largely differentiates lowland ecotypes from upland ecotypes, would be differentially expressed in leaves between the two ecotypes and/or carry NPs in the coding region that affected protein structure. Because we expected leaf wax to be fixed within an ecotype, we focused on NPs that were present in homozygous condition in both grandparents. Mining the database that we generated of annotated NPs and genes differentially expressed between AP13 and VS16, we identified 207 genes that carried homozygous variants between AP13 and VS16 in the coding region with predicted moderate to high impact effects on protein function, and 94 genes that had significantly different transcript levels in two lowland accessions (Alamo and Kanlow) compared to two upland accessions (Summer and Dacotah) (Supplementary Table S9). Of those, ten genes were common to the two classes. Gene ontology (GO) term analysis of the 207 genes with NPs in the coding region failed to identify any that were predicted to be involved in lipid metabolism. However, five of the 94 differentially expressed genes, Pavir.7KG097000, Pavir.7KG077981, Pavir.7KG013754, Pavir.7KG077009 and Pavir.7KG030500, had functional annotations and GO-terms indicative of putative involvement in wax synthesis (Supplementary Table S9). Pavir.7KG097000 and Pavir.7KG077981 had 92% identity over 85% of the protein length and had the highest homology (40% identity) in SwissProt (NCBI) to an (R)-specific enoyl-CoA hydratase from the bacterium *Aeromonas caviae*. Both genes are likely involved in fatty acid catabolism and were upregulated in the two upland accessions relative to the two lowland accessions. Pavir.7KG013754 and Pavir.7KG030500 were also duplicates (98% identity over 100% of the protein length) with 41% identity in SwissProt to acyl-acyl carrier protein (ACP) thioesterase ATL3 from *Arabidopsis*. The coding sequences of Pavir.7KG013754 and Pavir.7KG030500, extracted from the AP13 reference genome v5.1, were 648 bp in length, consisted of five exons and encoded 215 amino acid proteins. Both genes lacked expression in the uplands Summer and Dacotah in our RNASeq experiment. The fifth differentially expressed gene, Pavir.7KG077009, was annotated as a ‘chalcone and stilbene synthase, C-terminal domain/FAE1/Type III polyketide synthase-like protein’ in Phytozome (https://phytozome-next.jgi.doe.gov/). This 1425 bp gene lacked introns and encoded a 474 amino acid protein. A BLASTP analysis against NCBI’s SwissProt showed the Pavir.7KG077009 protein to have 51% identity (69% positives) over 98% of its length with *Arabidopsis* 3-ketoacyl-CoA synthase 5 (KCS-5). Pavir.7KG077009 was significantly higher expressed (log2-fold change of 9.5) in lowlands than in uplands in our RNASeq dataset (padj = 1.7*e*^−12^). The underlying read count showed that, similar to Pavir.7KG013754 and Pavir.7KG030500, Pavir.7KG077009 lacked expression in both Summer and Dacotah. The RNASeq results were confirmed by quantitative RT-PCR (Supplementary Figure S12). Visual inspection of the alignment of VS16 genome resequencing reads to the AP13 v5.1 reference genome showed that Pavir.7KG077009 as well as Pavir.7KG013754 and Pavir.7KG030500 were absent from VS16.

### Structural organization of the putative wax locus

**Fig. 4:**
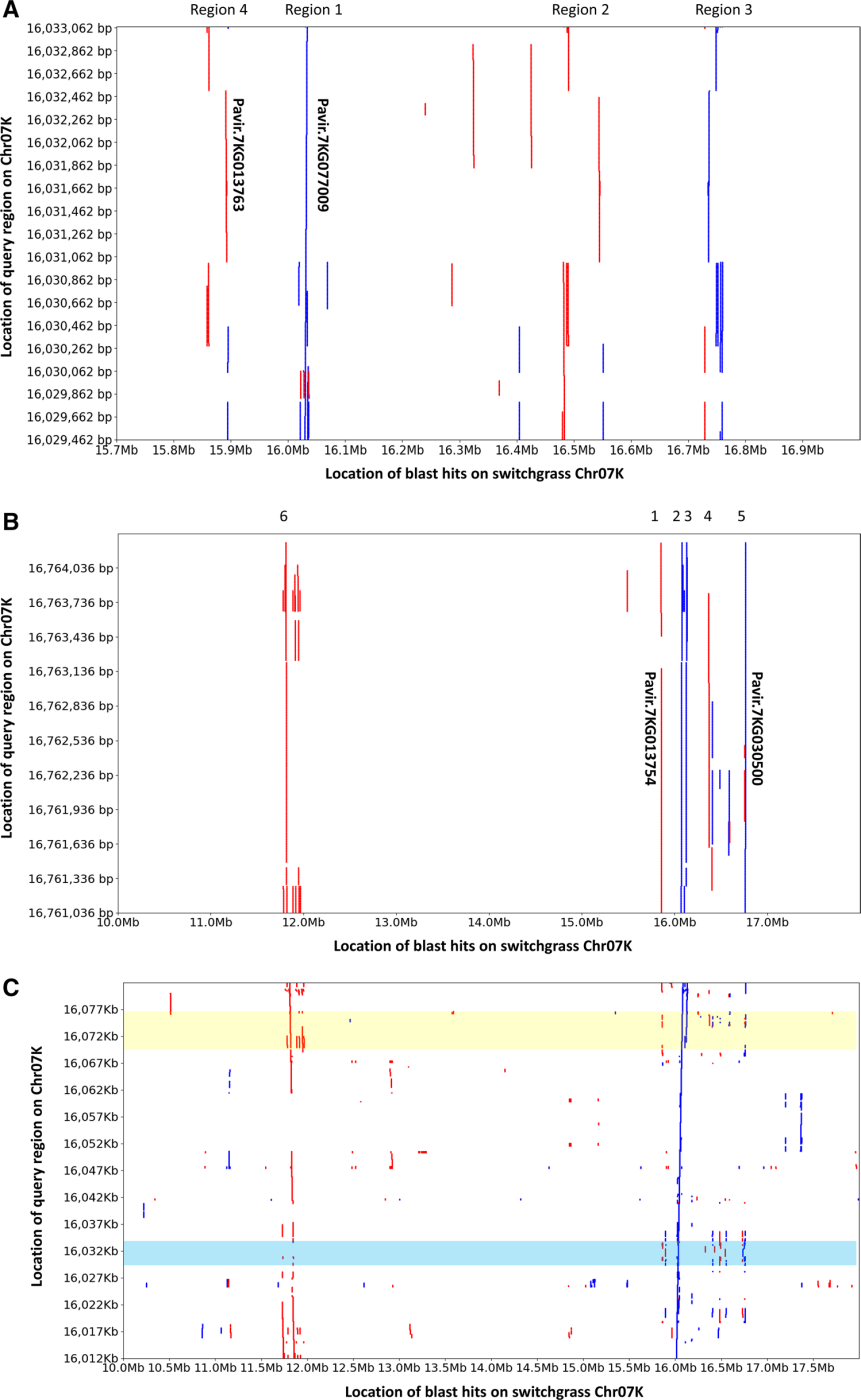
Graphical view of the BLASTN results on Chr07K using (**a**) Pavir.7KG077009 genomic sequence as query; (**b**) Pavir.7KG030500 genomic sequence as query; (**c**) an ~ 70 Kb region comprising Pavir.7KG077009 and the paralog of Pavir.7KG013754/Pavir.7KG030500 [indicated with ‘2’ in (**b**)] located most closely to Pavir.7KG077009; the region corresponding to Pavir.7KG077009 on the ~ 70 Kb query sequence and corresponding blast hits are highlighted in blue; the region corresponding to the paralog of Pavir.7KG013754/Pavir.7KG030500 on the ~ 70 Kb query sequence and the corresponding blast hits are highlighted in yellow. Blue lines indicate homology on the same DNA strand, and red lines indicate homology on the opposite DNA strand (color figure online)

No homoeologs of Pavir.7KG077009 and Pavir.7KG013754/Pavir.7KG030500 were found on Chr07N, despite an overall high level of homoeolog conservation between the switchgrass K and N genomes (Lovell et al. 2021). Interestingly, a BLASTN analysis using Pavir.7KG077009 showed that Chr07K carried a total of four regions (including Pavir.7KG077009) with high homology over more than 75% of the length of the Pavir.7KG077009 coding sequence as well as some smaller regions of homology (Fig. 4a). Similarly, using either Pavir.7KG013754 or Pavir.7KG030500 as a query in a BLASTN search against the switchgrass genome assembly identified a further four regions (in addition to Pavir.7KG013754 and Pavir.7KG030500) on Chr07K with high homology over more than 75% of the length of the Pavir.7KG013754/Pavir.7KG030500 coding sequence (Fig. 4b). All of the duplicated Pavir.7KG077009 gene copies and all but one of the duplicated Pavir.7KG013754/Pavir.7KG030500 gene copies were located in an approximately 1 Mb chromosome segment at location 15,854 Kb to 16,765 Kb, which we will refer to as the putative wax locus (Fig. 4a–c). The remaining copy of Pavir.7KG013754/Pavir.7KG030500 was located at 11,814–11,819 Kb. Of the replicated gene copies, only Pavir.7KG077009, Pavir.7KG013754 and Pavir.7KG030500 were full-length and uninterrupted by repetitive DNA. While the repeat patterns were complex and varied between the replicated regions, the fact that the same repeat(s) were found inserted at the same location into multiple pseudogene copies and that the repeat sequences flanking several pseudogenes also had high homology indicated that these regions originated through segmental duplications, possibly driven by transposable element (TE) activity. The largest paralogous gene fragment in region 4 (Fig. 4a) was annotated as Pavir.7KG013763 in the AP13 v5.1 reference genome, but is unlikely to yield a functional protein as it lacks approximately 200 amino acids at the N-terminus.

The many rearrangements that occurred in the putative wax region made it impossible for us to determine whether the Pavir.7KG013754/Pavir.7KG030500 duplications occurred largely as part of the same segmental duplications that amplified Pavir.7KG077009. However, this is likely the case. A BLASTN analysis of an ~ 70 Kb region (16,012 Kb–16,082 Kb) comprising Pavir.7KG077009 and one paralogous non-functional (due to the insertion of a CACTA transposable element in exon 1) copy of Pavir.7KG013754/Pavir.7KG030500 identified two highly homologous regions at ~ 11.73 Mb and ~ 11.86 Mb on Chr07K (Fig. 4c and Supplementary Figure S13A, B). Although the flanking sequences of Pavir.7KG077009 were present, the Pavir.7KG077009 paralog itself had been deleted from those regions (Supplementary Figure S13B), likely through illegitimate recombination as the deletion was flanked by two 8-bp repeats (1 bp mismatch) (Devos et al. 2002). However, a copy (also comprising the CACTA element in exon 1) of Pavir.7KG013754/Pavir.7KG030500 was present at ~ 11.82 Mb (Supplementary Figure S13A, C), supporting the occurrence of segmental duplications that included both Pavir.7KG013754/Pavir.7KG030500 and Pavir.7KG077009 paralogs. Alignment of the VS16 Illumina reads against the AP13 reference genome v5.1 indicated that both the 15,854 Kb–16,765 Kb region and the 11,750 Kb–11,858 Kb region were absent from VS16.

**Fig. 5:**
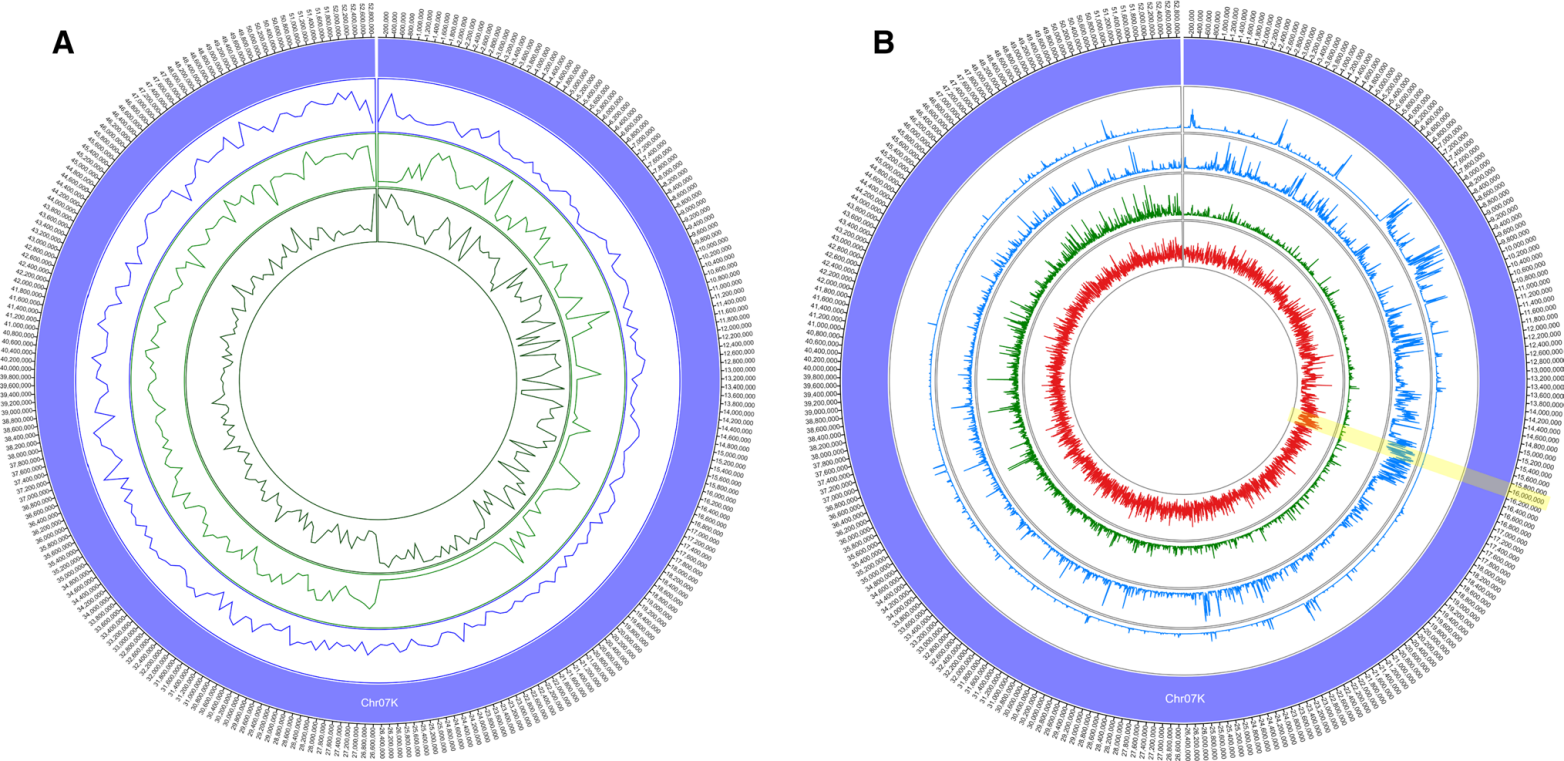
Circos diagrams showing **a** From outer to inner ring, the gene distribution on Chr07K (number of genes per 200 Kb, height min = 0, height max = 40), percentage of Chr07K genes with a homoeolog on switchgrass Chr07N (per 200 Kb, height min = 0, height max = 100), and percentage of Chr07K genes with a paralog in the switchgrass genome (excludes homoeologs) (per 200 Kb, height min = 0, height max = 100); **b** From outer to inner ring, the percentage of NPs that are missing in ≥ 50% of lowland accessions (per 10 Kb, height min = 0, height max = 100), percentage of NPs that are missing in ≥ 50% of upland accessions (per 10 Kb, height min = 0, height max = 100), the gene distribution (number of genes per 10 Kb, height min = 0, height max = 25), and the repeat distribution (number of repeats per 10 Kb, height min = 0, height max = 40). The wax region is highlighted in yellow (color figure online)

To investigate whether the wax region is located in a fast-evolving region of Chr07K, we considered the distribution of genes across Chr07K and whether these genes had homoeologs on Chr07N or paralogs elsewhere in the switchgrass genome. Overall gene density was significantly lower in the proximal 25 Mb (39.2 genes/Mb) compared to the distal 25 Mb (89.1 genes/Mb) of Chr07K. Furthermore, under the criteria used to establish homoeology, 77.8% of the genes in the proximal 25 Mb region, but only 40.1% of the genes in the distal 25 Mb region lacked a homoeolog on Chr07N. The genes in the proximal 25 Mb, however, were more than twice as likely (62.1% *vs.* 28.3%) to have a paralog elsewhere in the switchgrass genome compared to genes in the distal 25 Mb (Fig. 5a). Interestingly, the proximal 25 Mb also contained a higher percentage of NPs that were present in the AP13 (lowland) reference genome and absent in at least 50% of a set of 65 upland accessions (Fig. 5b). A total of 189 10 Kb regions were identified in the proximal 25 Mb of Chr07K in which at least 50% of the NPs had missing data in at least 50% of the upland accessions, but only 14 10 Kb regions with high numbers of missing NPs were identified in the distal 25 Mb of Chr07K. The number of 10 Kb regions with more than 50% of missing NPs in more than 50% of accessions in a set of 95 lowlands was 35 in the proximal 25 Mb and one in the distal 25 Mb of Chr07K. The 10 Kb regions with missing NPs often occurred in clusters, with the largest cluster containing the wax region (Fig. 5b).

Variant calling in the set of 95 lowland accessions (minor allele frequency ≥ 5%; missing data ≤ 30%) for Pavir.7KG077009, Pavir.7KG013754 and Pavir.7KG030500, as well as for Pavir.7KG013763, the truncated paralog to Pavir.7KG077009, identified no SNPs in Pavir.KG077009 (CDS length: 1425 bp; 1 exon), but seven synonymous SNPs, 18 non-synonymous SNPs and one nonsense mutation in Pavir.7KG013763, although the latter was present only in heterozygous condition in two accessions. While the accumulation of a larger number of mutations in a truncated and hence likely non-functional gene copy is expected, we saw a similar pattern in the two duplicated thioesterase-encoding genes. Two synonymous SNPs and no non-synonymous SNPs were identified in Pavir.7KG013754 (CDS length: 648 bp; 5 exons), while one high impact mutation (splice_acceptor_variant, heterozygous in three accessions), nine synonymous SNPs and 13 non-synonymous SNPs were present in 7KG030500 (CDS length: 648 bp; 5 exons).

## Discussion

### The genetic maps

Because switchgrass is an obligate outcrossing species, we generated F_2_ populations segregating for upland/lowland characteristics by first crossing the lowland (Alamo) genotype AP13 with the upland (Summer) genotype VS16 (Serba et al. 2013), followed by crossing two of the resulting F_1_ progeny. Across the two populations, an average of 67.1% of the markers were heterozygous in both F_1_ parents, 16.3% were heterozygous in the F_1_ used as female and homozygous in the F_1_ used as male parent, and 16.6% were homozygous in the female F_1_ and heterozygous in the male F_1_. Although we generated paternal (AH), maternal (HA) and HH linkage maps for each population, the HH linkage maps had the most comprehensive genome coverage, reflecting the high level of genetic diversity between switchgrass ecotypes. Although markers that were heterozygous in one parental F_1_ and homozygous in the other parental F_1_ were underrepresented in some chromosome regions, the fact that different regions were affected in the maternal and paternal maps and/or Pop1 and Pop2 (Fig. 1, Supplementary Figure S8) suggests that the lack of markers in the maternal and paternal maps was not due to fixation across large regions of different alleles in the lowland parent, AP13, and the upland parent, VS16. Possible exceptions are the distal region on Chr07K and interstitial region on Chr09N (Fig. 1). Fixation for alternate alleles in AP13 and VS16 in the distal region of Chr07K is supported by the fact that this region was also missing from the AP13 and VS16 maps that were generated in an AP13 × VS16 F_1_ population (Daverdin et al. 2015; Serba et al. 2013). Although the maternal and paternal maps generated from crosses between two F_1_ progeny have fewer markers than the HH maps generated in the same crosses, these maps may nevertheless be useful in mapping QTL for traits that are heterozygous in either one or both of the grandparents of the F_2_ progeny (AP13 and VS16 in our study).

High-quality linkage maps play a key role in the genetic analyses of traits. A high level of agreement between the linkage maps and the genome assembly is critical for identifying candidate genes underlying traits of interest. Very few discrepancies (~ 1.2%) were seen between marker positions in our linkage maps and switchgrass AP13 assembly v5.1. This is in contrast to an earlier assembly, v4.0, where some 28% of markers were located in non-colinear positions on the linkage map and the genome assembly. In fact, Pavir.7KG077009 and Pavir.7KG013754 were both lacking from the v4 assembly. Most of the discrepancies between the genetic maps and genome assemblies were discerned by both the Pop1 and Pop2 linkage maps, demonstrating the robustness of the maps.

### Leaf wax

Cuticular wax plays an important role in the abiotic and biotic stress tolerance of plants (Gorb and Gorb 2017; Guo et al. 2016; Smith et al. 2006; Zhou et al. 2013). Lowland ecotypes of switchgrass are characterized by a bluish tint caused by the presence of rod-shaped wax crystals on the abaxial leaf side, while upland ecotypes are non-glaucous (Bragg et al. 2020; Weaver et al. 2018). An analysis of the composition of leaf wax in switchgrass showed that the predominant components are C33 β-diketones (Bragg et al. 2020; Tulloch and Hoffman 1980; Weaver et al. 2018), similar to what has been observed in a variety of other species including wheat, barley, *Rhododendron* and *Hosta* (Evans et al. 1975; Hen-Avivi et al. 2016; Jenks et al. 2002). *Arabidopsis*, however, does not produce β-diketone cuticular wax (Kosma and Rowland 2016). A comparison of the wax content and composition of glaucous lowlands *vs.* non-glaucous variants of the lowland switchgrass accession Alamo and glossy uplands revealed that the chemical components most affected were the β-diketones, which were reduced, depending on the study, from 18 to 36% to less than 2% of the epicuticular wax content in non-glaucous genotypes (Bragg et al. 2020; Weaver et al. 2018).

Recent work in the *Triticeae* species wheat and barley suggests that β-diketones are synthesized through the concerted action of proteins encoded by co-expressed members of three gene families, a type-III polyketide synthase (PKS), a hydrolase and a cytochrome P450, that are present in a metabolic cluster on chromosome 2 (Hen-Avivi et al. 2016). The proposed pathway involves conversion by the hydrolase of plastid-produced 3-ketoacyl-ACP (acyl carrier protein) to 3-ketoacid, which is then further converted by the type-III PKS to β-diketone. The P450 hydroxylates β-diketone to hydroxy-β-diketone (Hen-Avivi et al. 2016).

We identified large effect QTL for both the visual scoring of the presence of wax and for the abaxial hydrophobicity measurements taken at different distances from the base of the leaf blade on chromosome 7K (Fig. 3) that explained, on average across replicates and measurements, 28% of the phenotypic variation (Table 2). A QTL for surface wax on Chr07K was reported in the region 33.0–35.8 Mb by Bragg and colleagues (Bragg et al. 2020). This QTL was adjacent to, but did not overlap with the wax QTL identified in our study (0–31 Mb). The two factors that were fundamentally different between the two studies were the mapping population (four-way cross of which two parents were common to our study) and the methodology used for measuring wax load. Bragg et al. (2020) extracted leaf cuticular wax from both sides of the leaf while we specifically phenotyped for wax level on the abaxial leaf side. Both Weaver et al. (2018) and Bragg et al. (2020) have shown that the structure of the wax crystals, and hence likely the composition of the wax, differed on the adaxial and abaxial leaf sides. Although we did not measure hydrophobicity levels on the adaxial leaf side across the entire population, a small-scale study on AP13, VS16 and nine F_2_ progeny showed considerably lower variation in adaxial hydrophobicity levels across the samples (Supplementary Table S6), and uncorrelated adaxial and abaxial measurements (Supplementary Table S7). We surmise that using combined adaxial and abaxial wax load as a phenotype as opposed to only abaxial wax levels may have resulted in the different QTL locations in the two studies.

Although the Chr07K QTL interval identified in our study for abaxial wax spanned 31 Mb, this region was relatively gene-poor (Fig. 5a). Nevertheless, the 0–31 Mb region comprised 1377 genes in the AP13 reference assembly v5.1 of which 810 were functionally annotated. Five of these genes were differentially expressed in the two lowland cultivars compared to the two upland cultivars tested and had either ‘fatty acid biosynthetic process’ or ‘lipid metabolic process’ as an associated GO term. Pavir.7KG097000 and Pavir.7KG077981 were upregulated in the two upland accessions relative to the two lowland accessions and are likely involved in fatty acid catabolism. Hence, they were unlikely to be causal to the wax QTL on Chr07K.

The proteins encoded by the duplicated genes Pavir.7KG013754 and Pavir.7KG030500 had the highest homology in SwissProt to *Arabidopsis* ATL3. ALT3 is plastid located, and heterologous expression in *E. coli* indicated that it likely converts fatty acyl-ACP to β-keto acids (Pulsifer et al. 2014). Although structurally distinct from the acyl-ACP thioesterase FATB, ALT3 may be partially redundant in function with FATB (Pulsifer et al. 2014). Knockout of *fatb* in *Arabidopsis* led to a reduction in wax load, but not wax composition, supporting FATB’s role in providing wax precursors (Bonaventure et al. 2003). Based on the function of *Arabidopsis* ALT3 and of the related protein methylketone synthase 2 (ShMKS2) in wild tomato (Pulsifer et al. 2014; Yu et al. 2010), the acyl-ACP thioesterases Pavir.7KG013754 and Pavir.7KG030500 may catalyze the hydrolysis from 3-ketoacyl-ACP to 3-ketoacids in plastids. The putative function of Pavir.7KG013754 and Pavir.7KG030500, and their absence in the uplands Dacotah and Summer led us to hypothesize that these duplicated genes may play a role in determining the overall wax load in switchgrass.

Pavir.7KG077009 encodes a putative 3-ketoacyl-CoA synthase 5 (KCS-5, synonym eciferum 60 (CER60)). A Type III polyketide synthase has been shown to play a role in β-diketone synthesis in the *Triticeae* species wheat and barley (Hen-Avivi et al. 2016), and hence, Pavir.7KG077009, which conceivably converts 3-ketoacids to β-diketones, was considered a likely candidate controlling β-diketone levels in switchgrass cuticular wax. Furthermore, Pavir.7KG077009 was absent in the two upland accessions tested. Loss of function mutants of *kcs5* in pennycress (*Thlaspi arvense*) lack very long chain fatty acids (VLCFAs), which results in a waxless phenotype (Chopra et al. 2018). Related *kcs* genes such as *cer6* in *Arabidopsis* and *wsl4* (*wax crystal-sparse leaf 4*) in rice have also been shown to play an essential role in wax production (Hooker et al. 2002; Wang et al. 2017). Neither the putative acyl-ACP thioesterases nor KCS-5 have a homoeolog on chromosome 7N, so the absence of both genes in uplands would reduce both the overall wax load and the β-diketone content.

The proteins encoded by Pavir.7KG013754/Pavir.7KG030500 and Pavir.7KG077009 appear to have similar functions to the barley hydrolase and type-III polyketide that constitute the *cer-cqu* locus, although reciprocal BLASTP analyses indicated that they were not orthologous, and had only limited homology (MLOC_59804 and Pavir.7KG077009, < 30% identity over less than 25% of the protein length) or no homology (Pavir.7KG013754/Pavir.7KG030500) to the barley *cer-cqu* proteins. The *cer-cqu* locus contains a third gene involved in the control of leaf wax, a cytochrome P450 that converts β-diketone to hydroxy-β-diketone (Hen-Avivi et al. 2016). Interestingly, a cytochrome P450 (Pavir.7KG037800) was also found clustered with Pavir.7KG077009, Pavir.7KG013754 and Pavir.7KG030500. Pavir.7KG037800 is neither orthologous nor paralogous to the barley CER-U P450 and has no associated GO-terms, so it is unknown whether it is involved in fatty acid biosynthesis. If the putative KCS-5 and acyl-ACP esterases and, potentially, the cytochrome P450, all of which are absent from the uplands Summer and Dacotah, are indeed involved in the β-diketone pathway, it is possible that these genes need to be co-expressed, which could be facilitated by their colocalization. A number of gene clusters, consisting of genes that are involved in the same pathway but unrelated in sequence, have been identified in plants (Osbourn 2010). CRISPR-Cas9 knockout of Pavir.7KG077009, Pavir.7KG013754 and Pavir.7KG030500 in the Alamo-derived transformable genotype ‘Performer’ has been initiated.

### Evolution of the wax region in switchgrass

Pavir.7KG077009 and Pavir.7KG013754/Pavir.7KG030500 only had around 62% and 65% of identity at the protein level to their closest homologs in switchgrass and other grass species, respectively, which indicates their potentially unique function. The lack of homoeologs and syntenic orthologs suggests that Pavir.7KG077009 and Pavir.7KG013754/Pavir.7KG030500 most likely originated through duplication from genes elsewhere in the genome. We were, however, unable to establish the identity of the ‘mother copies'. Nevertheless, none of the distant homologs of KCS5 and acyl-ACP thioesterases colocalized, suggesting that both genes were duplicated to the same region of Chr07K independently and then underwent rapid neofunctionalization. Several lines of evidence point at the fact that the Chr07K region that contains the putative β-diketone pathway genes is fast evolving. The region is repeat-rich and gene-poor, and enriched for genes that lack a homoeolog on Chr07N in the AP13 reference genome assembly v5.1 (Fig. 5a). Interestingly, this region also has hotspots of deletions, particularly in upland accessions, relative to the AP13 reference genome with Pavir.7KG077009 and Pavir.7KG013754/Pavir.7KG030500 being located in the largest of the deletion hotspots (Fig. 5b). Despite the rapid evolution of this region of switchgrass Chr07K, including complete or partial deletion and inactivation through insertion of transposable elements of Pavir.7KG077009 and Pavir.7KG013754/Pavir.7KG030500 paralogs, at least one full-length copy of each of these genes has been retained. The lack of any amino acid substitutions in the proteins encoded by Pavir.7KG077009 and Pavir.7KG013754, and the accumulation of mutations in their paralogs, suggests that the presence of one functional gene copy each for a putative KCS protein and acyl-ACP thioesterase confers a selective advantage in lowland accessions.

## Conclusions

In this study, we have demonstrated how a combination of next-generation sequencing tools and resources can be employed to predict strong gene candidates for trait QTL. Our workflow consisted of (1) using GBS to genotype the mapping population and generate high-quality linkage maps; (2) conducting QTL analyses and projecting the QTL regions on a high-quality genome assembly; (3) generating a database of NP variants between the (grand)parents of the mapping population from resequencing data; and (4) generating and mining transcriptome data for the two parents to identify genes that were differentially expressed between the mapping (grand)parents. Our work led to the identification of a FAE1/Type III polyketide synthase-like protein and two copies of an acyl-ACP thioesterase, all of which are unique in the switchgrass genome and likely originated through duplication followed by neofunctionalization, as strong candidate genes for the control of leaf glaucousness in lowland switchgrass. While this study was focused on identifying the gene(s) that controls β-diketone wax production, the established workflow and resources, supplemented with transcriptome data for additional tissues, developmental stages and varying stress conditions, can be applied to other traits mapped in the same population.

## Supplementary Information

Below is the link to the electronic supplementary material.

**Figure S1.** The distribution of, from outer to inner ring, NPs (SNPs and InDels) between AP13 and VS16 identified from GBS data (per Mb, height min = 0, height max = 3,600), annotated genes in AP13 assembly v5.1 (per Mb, height min = 0, height max = 160), and NP markers mapped in the Pop1 HH map and the Pop2 HH map (per Mb, height min = 0, height max = 30). The colored blocks indicate the chromosomes as represented in switchgrass AP13 assembly v5.1.

Supplementary Information 1 (PDF 594 kb)

**Figure S2.** HH genetic map comprised of markers that were heterozygous in both parents from Pop1. Only one marker per set of cosegregating markers is shown.

Supplementary Information 2 (PDF 4334 kb)

**Figure S3.** HH genetic map comprised of markers that were heterozygous in both parents from Pop2. Only one marker per set of cosegregating markers is shown.

Supplementary Information 3 (PDF 2672 kb)

**Figure S4.** Paternal (AH) genetic map of Pop1. Only one marker per set of cosegregating markers is shown.

Supplementary Information 4 (PDF 622 kb)

**Figure S5.** Paternal (AH) genetic map of Pop2. Only one marker per set of cosegregating markers is shown.

Supplementary Information 5 (PDF 1091 kb)

**Figure S6.** Maternal (HA) genetic map of Pop1. Only one marker per set of cosegregating markers is shown.

Supplementary Information 6 (PDF 1132 kb)

**Figure S7.** Maternal (HA) genetic map of Pop2. Only one marker per set of cosegregating markers is shown.

Supplementary Information 7 (PDF 661 kb)

**Figure S8. A-B:** Dot plot comparisons of AP13 genome assembly v5.1 with the maternal (pink dots), paternal (blue dots) and HH (black dots) linkage maps of Pop1 (**A**) and Pop2 (**B**). **C-F**: Circos diagrams showing the relationships between AP 13 genome assembly v5.1 and the Pop1 paternal map (**C**), Pop1 maternal map (**D**), Pop2 paternal map (**E**) and Pop2 maternal map (**F**).

Supplementary Information 8 (PDF 4249 kb)

**Figure S9.** Circos diagrams showing the relationships of the genome assemblies of *Sorghum bicolor*, *Oryza sativa*, *Setaria italica*, and *Brachypodium distachyon* with the switchgrass HH linkage maps generated in Pop1 (**A**) and Pop2 (**B**).

Supplementary Information 9 (PDF 666 kb)

**Figure S10.** Circos diagram showing the distribution of SNPs (outer ring) and InDels (inner ring) identified between AP13 and VS16 from resequencing data across the AP13 genome assembly v5.1.

Supplementary Information 10 (PDF 654 kb)

**Figure S11.** Graphs showing (**A**) drop contact angles at different positions from the base of the leaf blade and (**B**) visually scored wax level on the abaxial leaf surface for lines carrying the AP13 (A), VS16 (B) and H alleles at marker Tag_7893. The droplet contact angle means were calculated across 145 progeny; Mean wax levels were calculated across 330 progeny.

Supplementary Information 11 (PDF 127 kb)

**Figure S12.** Expression analysis of Pavir.7KG077009 in Alamo, Dacotah, Kanlow and Summer by quantitative RT-PCR.

Supplementary Information 12 (PDF 85 kb)

**Figure S13.** Graphical view of the BLASTN results on Chr07K using (**A**) an ~ 70 Kb region comprising Pavir.7KG077009 and the paralog of Pavir.7KG013754/Pavir.7KG030500 (indicated with ‘2’ in Fig. 4B) located most closely to Pavir.7KG077009; the region corresponding to Pavir.7KG077009 on the ~ 70 Kb query sequence and corresponding blast hits are highlighted in blue; the region corresponding to the paralog of Pavir.7KG013754/Pavir.7KG030500 on the ~ 70 Kb query sequence and the corresponding blast hits are highlighted in yellow. (**B**) Detailed view of region indicated with dashed blue box in (A) showing the presence of regions homologous to the flanking regions of Pavir.7KG077009, but absence of homology to Pavir.7KG077009 itself (corresponding region is highlighted in blue). (**C**) Detailed view of the dashed black box in (A) showing the presence of homology to Pavir.7KG013754/Pavir.7KG030500 (corresponding region is highlighted in yellow). Blue lines indicate homology on the same DNA strand, red lines indicate homology on the opposite DNA strand.

Supplementary Information 13 (PPTX 217 kb)

**Table S1.** Accessions together with their ecotype and subpopulation membership (from Lovell et al. 2021) used for nucleotide deletion analysis.

Supplementary Information 14 (XLSX 12 kb)

**Table S2.** Paternal (AH), maternal (HA) and HH linkage maps of Pop1 and Pop2 generated with GBS-NPs identified using switchgrass AP13 genome assembly v2.0 (AP13V2) as reference, and the location of these NPs on switchgrass AP13 assembly v4.0 (AP13V4), switchgrass AP13 assembly v5.1 (AP13V51), *Brachypodium distachyon* assembly v3.0 (bdi), *Oryza sativa* assembly v7.0 (os), *Sorghum bicolor* assembly v3.1.1 (sbi), and *Setaria italica* assembly v2.2 (si).

Supplementary Information 15 (XLSX 24055 kb)

**Table S3.** The NPs and their flanking sequence.

Supplementary Information 16 (XLSX 4834 kb)

**Table S4.** Classification of NPs identified between AP13 and VS16 by region, impact and functional class.

Supplementary Information 17 (XLSX 10 kb)

**Table S5.** Analysis of variance of droplet contact angles measured on the abaxial and adaxial leaf surfaces across AP13, VS16 and nine randomly selected F_2_ progeny.

Supplementary Information 18 (XLSX 10 kb)

**Table S6.** Contact angle measurements on the adaxial (A) and abaxial (B) leaf side at different locations from the leaf base for the grandparents (AP13 and VS16) and nine randomly selected F_2_ progeny.

Supplementary Information 19 (XLSX 10 kb)

**Table S7.** Pearson’s correlation coefficients of the droplet contact angles on the adaxial leaf side and those on the abaxial leaf side, and the corresponding p-values of the correlations, generated using the rcorr function in the Hmisc package in R version 3.2.2.

Supplementary Information 20 (XLSX 8 kb)

**Table S8.** Droplet contact angles (‘droplet contact angle’ tab) and visual wax scores (‘wax’ tab) for Pop2 progeny. The presence of wax on the abaxial leaf side was assessed visually on three replicates of the 330 F_2_ progeny of Pop2 in the field using a scoring scale from 1 to 4, whereby 1 refers to a glossy green color (low wax level) and 4 refers to dull bluish color (high wax level).

Supplementary Information 21 (XLSX 25 kb)

**Table S9.** Genes in the region 0–31 Mb on chromosome 7K that are (1) differentially expressed between the lowland accessions Alamo and Kanlow, and the upland accessions Summer and Dacotah ('DE genes' tab); (2) carry NPs that differentiate AP13 from VS16 with predicted moderate to high effects on protein function ('HighModerate NP effect genes' tab).

Below is the link to the electronic supplementary material.

Supplementary Information 22 (XLSX 87 kb)

## Data Availability

All scripts used have previously been published and have been referenced.
