# Supplementary material for: Quantitative trait locus mapping combined with variant and transcriptome analyses identifies a cluster of gene candidates underlying the variation in leaf wax between upland and lowland switchgrass ecotypes
Source: Theor Appl Genet. 2021 Mar 24;134(7):1957–75. doi: 10.1007/s00122-021-03798-y (PMC8263549; doi:10.1007/s00122-021-03798-y)
Supplement: Supplementary file 1 — Supplementary Information 1 (PDF 594 kb) [file 122_2021_3798_MOESM1_ESM.pdf]

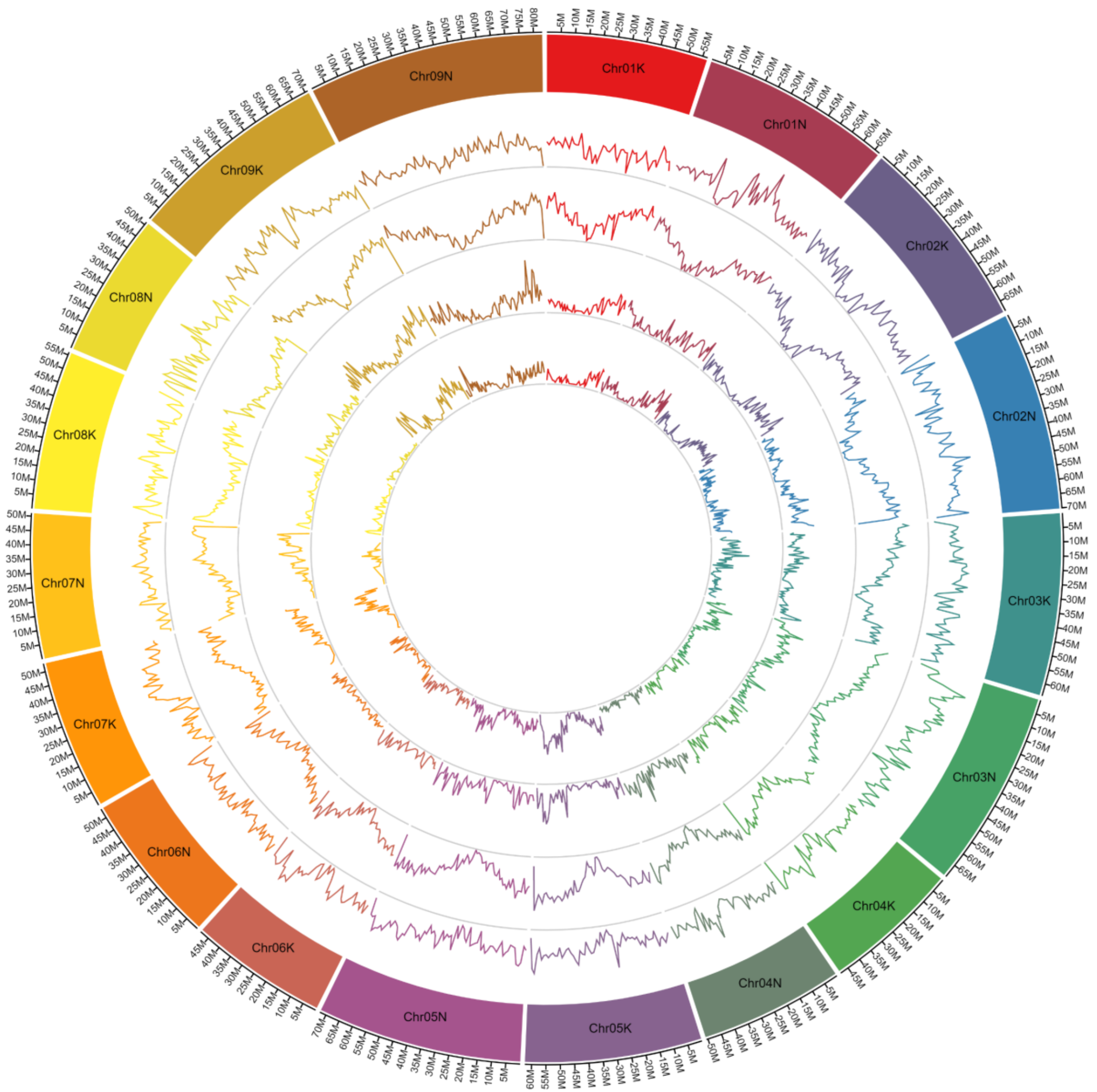

Figure S1. The distribution of, from outer to inner ring, NPs (SNPs and InDels) between AP13 and VS16 identified from GBS data (per Mb, height min=0, height max=3,600), annotated genes in AP13 assembly v5.1 (per Mb, height min=0, height max=160), and NP markers mapped in the Pop1 HH map and the Pop2 HH map (per Mb, height min=0, height max=30). The colored blocks indicate the chromosomes as represented in switchgrass AP13 assembly v5.1.
