# Supplementary material for: Quantitative trait locus mapping combined with variant and transcriptome analyses identifies a cluster of gene candidates underlying the variation in leaf wax between upland and lowland switchgrass ecotypes
Source: Theor Appl Genet. 2021 Mar 24;134(7):1957–75. doi: 10.1007/s00122-021-03798-y (PMC8263549; doi:10.1007/s00122-021-03798-y)
Supplement: Supplementary file 3 — Supplementary Information 3 (PDF 2672 kb) [file 122_2021_3798_MOESM3_ESM.pdf]

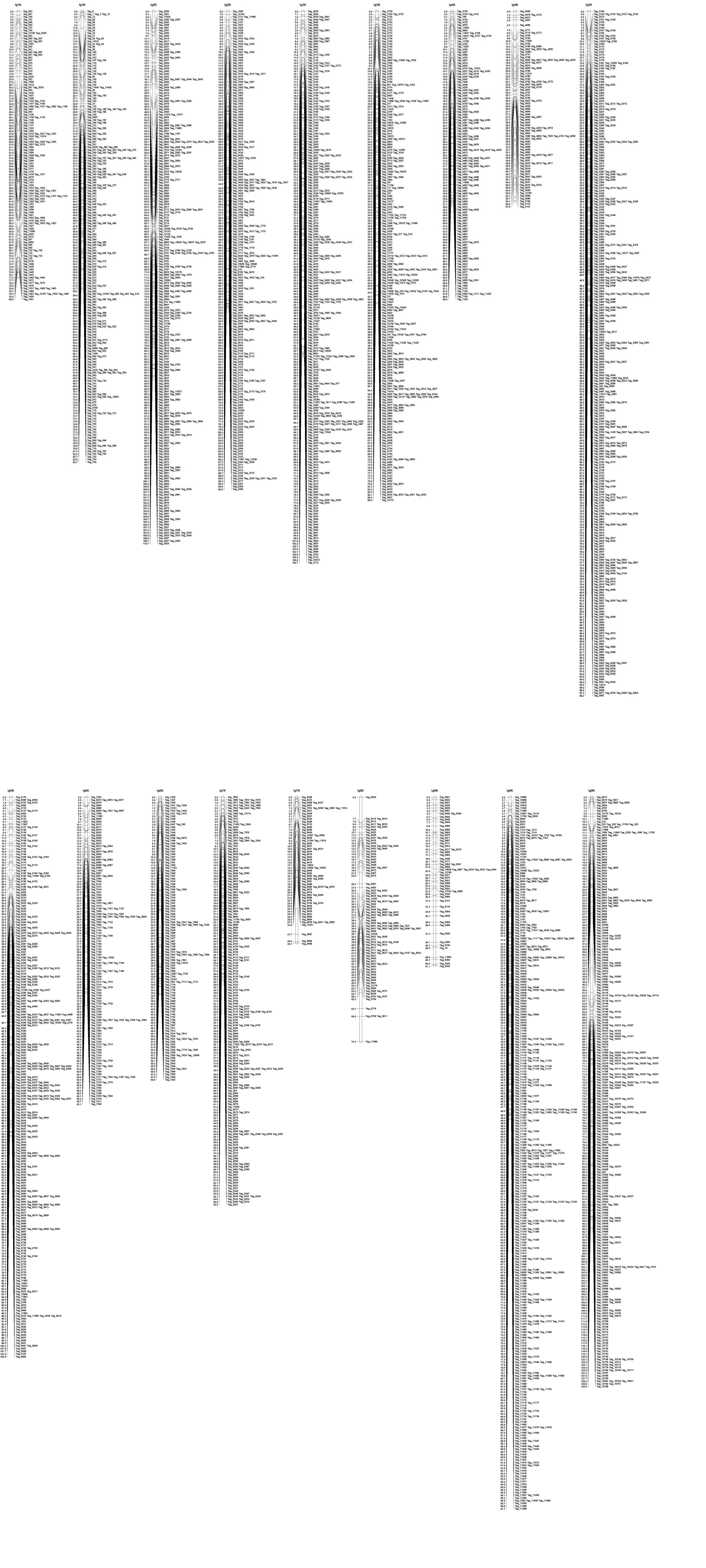

Figure S3. HH genetic map comprised of markers that were heterozygous in both parents from Pop2. Only one marker per set of cosegregating markers is shown.
