# Supplementary figures and images for: Quantitative trait locus mapping combined with variant and transcriptome analyses identifies a cluster of gene candidates underlying the variation in leaf wax between upland and lowland switchgrass ecotypes
Source: Theor Appl Genet. 2021 Mar 24;134(7):1957–75. doi: 10.1007/s00122-021-03798-y (PMC8263549; doi:10.1007/s00122-021-03798-y)

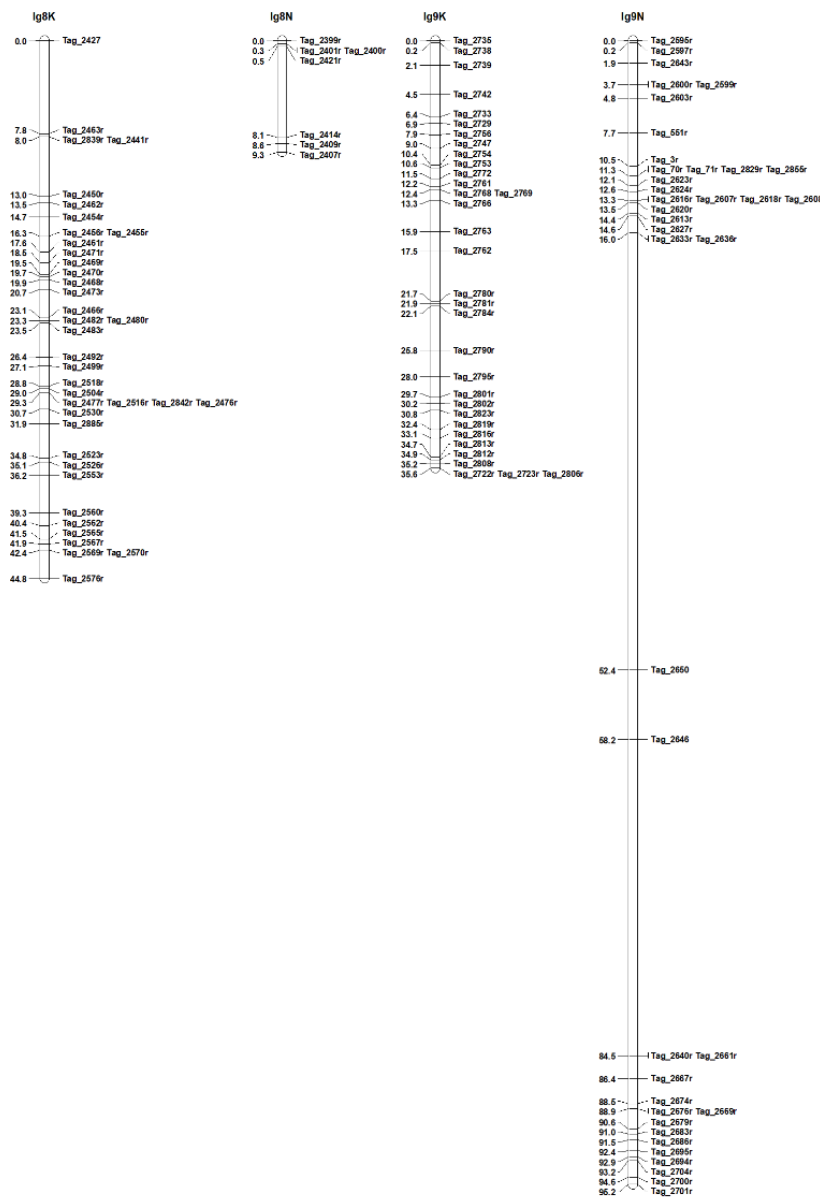

Supplement: Supplementary file 6 — Supplementary Information 6 (PDF 1132 kb) [file 122_2021_3798_MOESM6_ESM.pdf]
