# Supplementary material for: Quantitative trait locus mapping combined with variant and transcriptome analyses identifies a cluster of gene candidates underlying the variation in leaf wax between upland and lowland switchgrass ecotypes
Source: Theor Appl Genet. 2021 Mar 24;134(7):1957–75. doi: 10.1007/s00122-021-03798-y (PMC8263549; doi:10.1007/s00122-021-03798-y)
Supplement: Supplementary file 7 — Supplementary Information 7 (PDF 661 kb) [file 122_2021_3798_MOESM7_ESM.pdf]

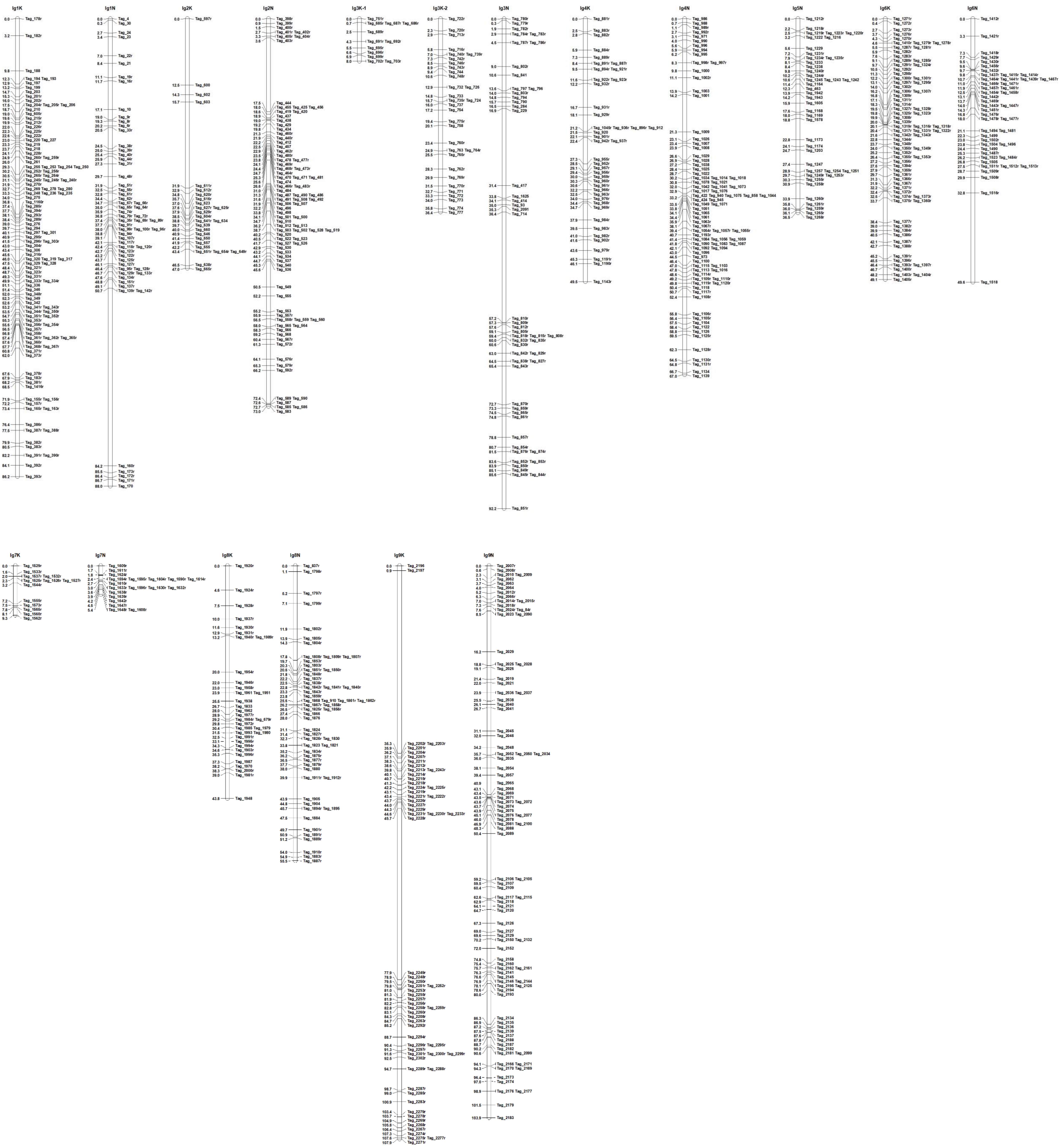

Figure S7. Maternal (HA) genetic map of Pop2. Only one marker per set of cosegregating markers is shown.
