# Supplementary material for: Quantitative trait locus mapping combined with variant and transcriptome analyses identifies a cluster of gene candidates underlying the variation in leaf wax between upland and lowland switchgrass ecotypes
Source: Theor Appl Genet. 2021 Mar 24;134(7):1957–75. doi: 10.1007/s00122-021-03798-y (PMC8263549; doi:10.1007/s00122-021-03798-y)
Supplement: Supplementary file 8 — Supplementary Information 8 (PDF 4249 kb) [file 122_2021_3798_MOESM8_ESM.pdf]

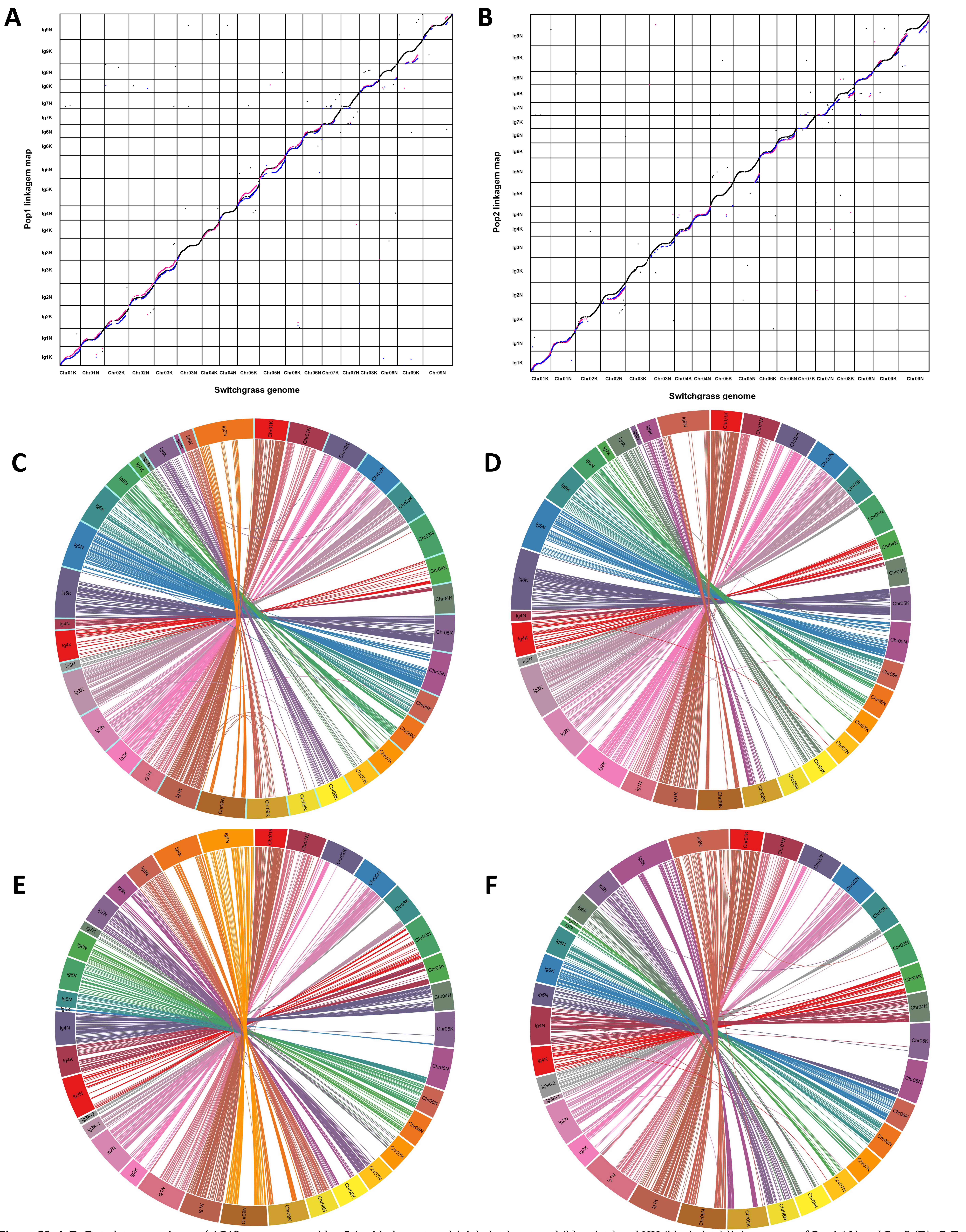

Figure S8. A-B. Dot plot comparisons of AP13 genome assembly v5.1 with the maternal (pink dots), paternal (blue dots) and HH (black dots) linkage maps of Pop1 (**A**) and Pop2 (**B**). **C-F**: Circos diagrams showing the relationships between AP 13 genome assembly v5.1 and the Pop1 paternal map (**C**), Pop1 maternal map (**D**), Pop2 paternal map (**E**) and Pop2 maternal map (**F**).
