# Supplementary material for: Quantitative trait locus mapping combined with variant and transcriptome analyses identifies a cluster of gene candidates underlying the variation in leaf wax between upland and lowland switchgrass ecotypes
Source: Theor Appl Genet. 2021 Mar 24;134(7):1957–75. doi: 10.1007/s00122-021-03798-y (PMC8263549; doi:10.1007/s00122-021-03798-y)
Supplement: Supplementary file 9 — Supplementary Information 9 (PDF 666 kb) [file 122_2021_3798_MOESM9_ESM.pdf]

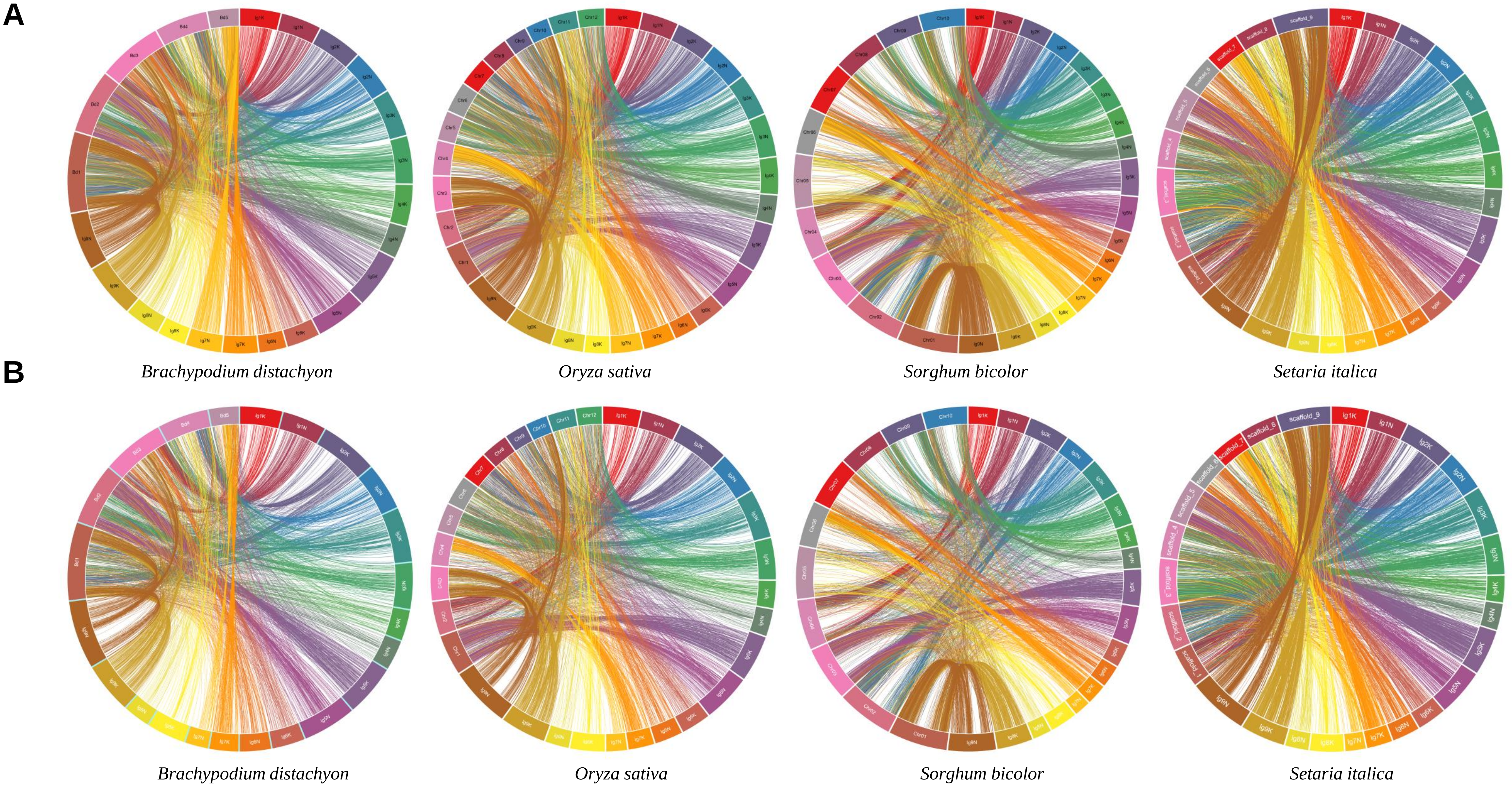

Figure S9. Circos diagrams showing the relationships of the genome assemblies of *Sorghum bicolor*, *Oryza sativa*, *Setaria italica*, and *Brachypodium distachyon* with the switchgrass HH linkage maps generated in Pop1 (**A**) and Pop2 (**B**).
