# Supplementary material for: Quantitative trait locus mapping combined with variant and transcriptome analyses identifies a cluster of gene candidates underlying the variation in leaf wax between upland and lowland switchgrass ecotypes
Source: Theor Appl Genet. 2021 Mar 24;134(7):1957–75. doi: 10.1007/s00122-021-03798-y (PMC8263549; doi:10.1007/s00122-021-03798-y)
Supplement: Supplementary file 10 — Supplementary Information 10 (PDF 654 kb) [file 122_2021_3798_MOESM10_ESM.pdf]

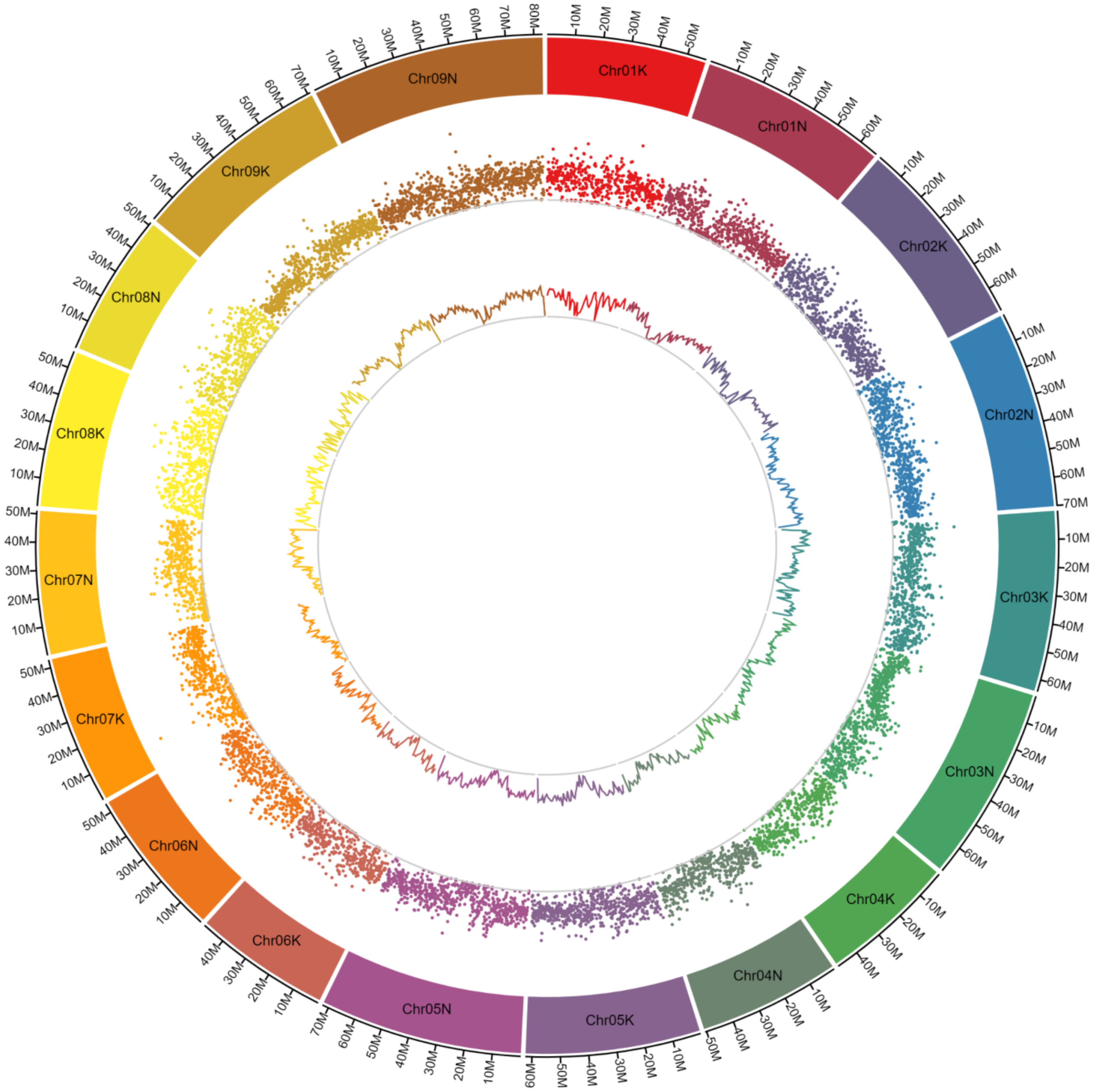

Figure S10. Circos diagram showing the distribution of SNPs (outer ring) and InDels (inner ring) identified between AP13 and VS16 from resequencing data across the AP13 genome assembly v5.1.
