# Supplementary material for: Quantitative trait locus mapping combined with variant and transcriptome analyses identifies a cluster of gene candidates underlying the variation in leaf wax between upland and lowland switchgrass ecotypes
Source: Theor Appl Genet. 2021 Mar 24;134(7):1957–75. doi: 10.1007/s00122-021-03798-y (PMC8263549; doi:10.1007/s00122-021-03798-y)
Supplement: Supplementary file 11 — Supplementary Information 11 (PDF 127 kb) [file 122_2021_3798_MOESM11_ESM.pdf]

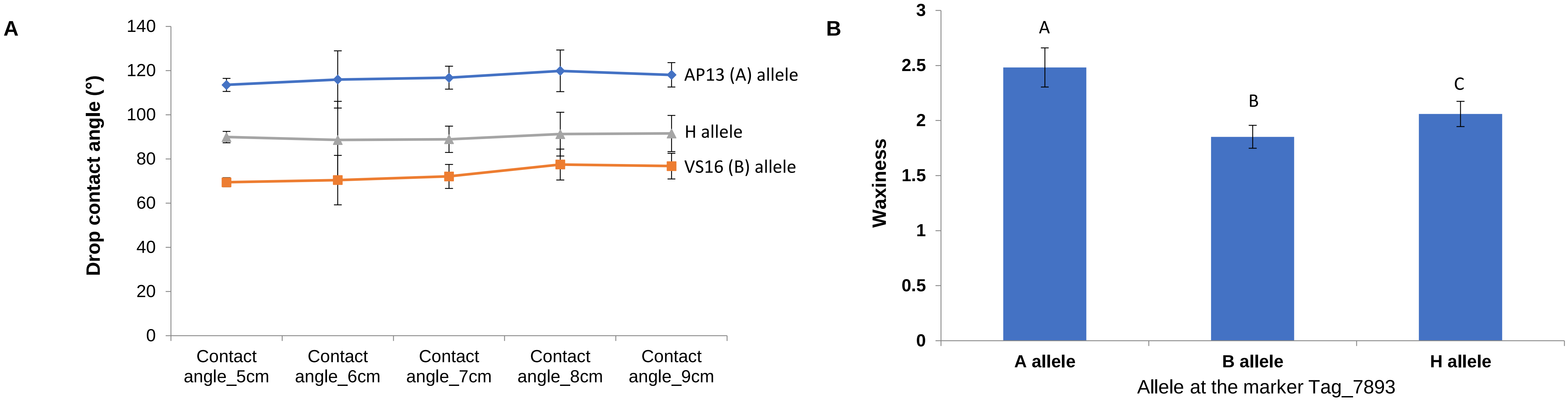

Figure S11. Graphs showing **(A)** drop contact angles at different positions from the base of the leaf blade and **(B)** visually scored wax level on the abaxial leaf surface for lines carrying the AP13 (A), VS16 (B) and H alleles at marker Tag_7893. The droplet contact angle means were calculated across 145 progeny; Mean wax levels were calculated across 330 progeny.
