# Supplementary material for: Quantitative trait locus mapping combined with variant and transcriptome analyses identifies a cluster of gene candidates underlying the variation in leaf wax between upland and lowland switchgrass ecotypes
Source: Theor Appl Genet. 2021 Mar 24;134(7):1957–75. doi: 10.1007/s00122-021-03798-y (PMC8263549; doi:10.1007/s00122-021-03798-y)
Supplement: Supplementary file 12 — Supplementary Information 12 (PDF 85 kb) [file 122_2021_3798_MOESM12_ESM.pdf]

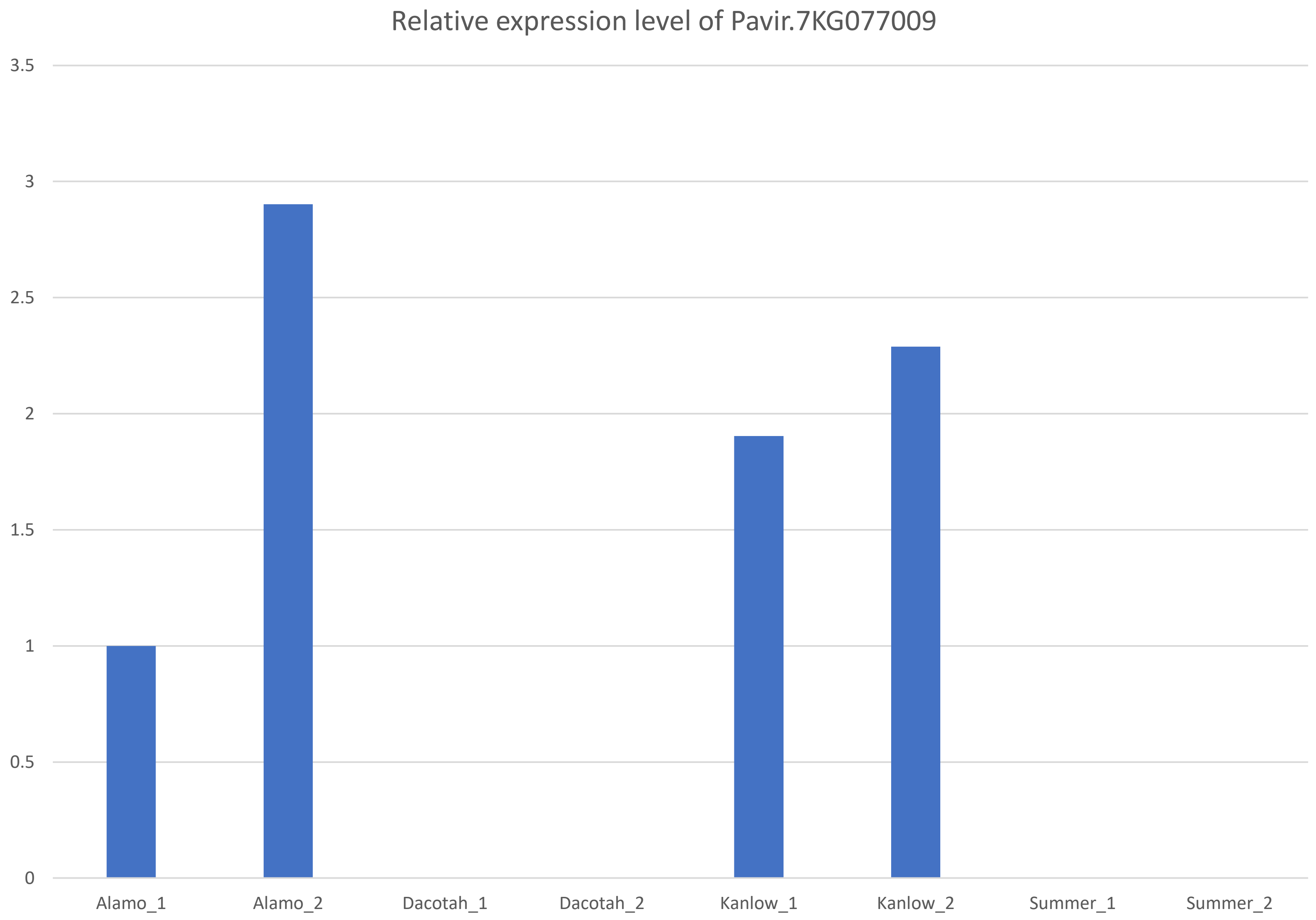

Figure S12. Expression analysis of Pavir.7KG077009 in two replicates each of Alamo (lowland), Dacotah (upland), Kanlow (lowland) and Summer (upland) by quantitative RT-PCR (qRT-PCR). Expression values are relative to the expression in Alamo_1.
