# Supplementary material for: Quantitative trait locus mapping combined with variant and transcriptome analyses identifies a cluster of gene candidates underlying the variation in leaf wax between upland and lowland switchgrass ecotypes
Source: Theor Appl Genet. 2021 Mar 24;134(7):1957–75. doi: 10.1007/s00122-021-03798-y (PMC8263549; doi:10.1007/s00122-021-03798-y)
Supplement: Supplementary file 13 — Supplementary Information 13 (PPTX 217 kb) [file 122_2021_3798_MOESM13_ESM.pptx]

## Slide 1
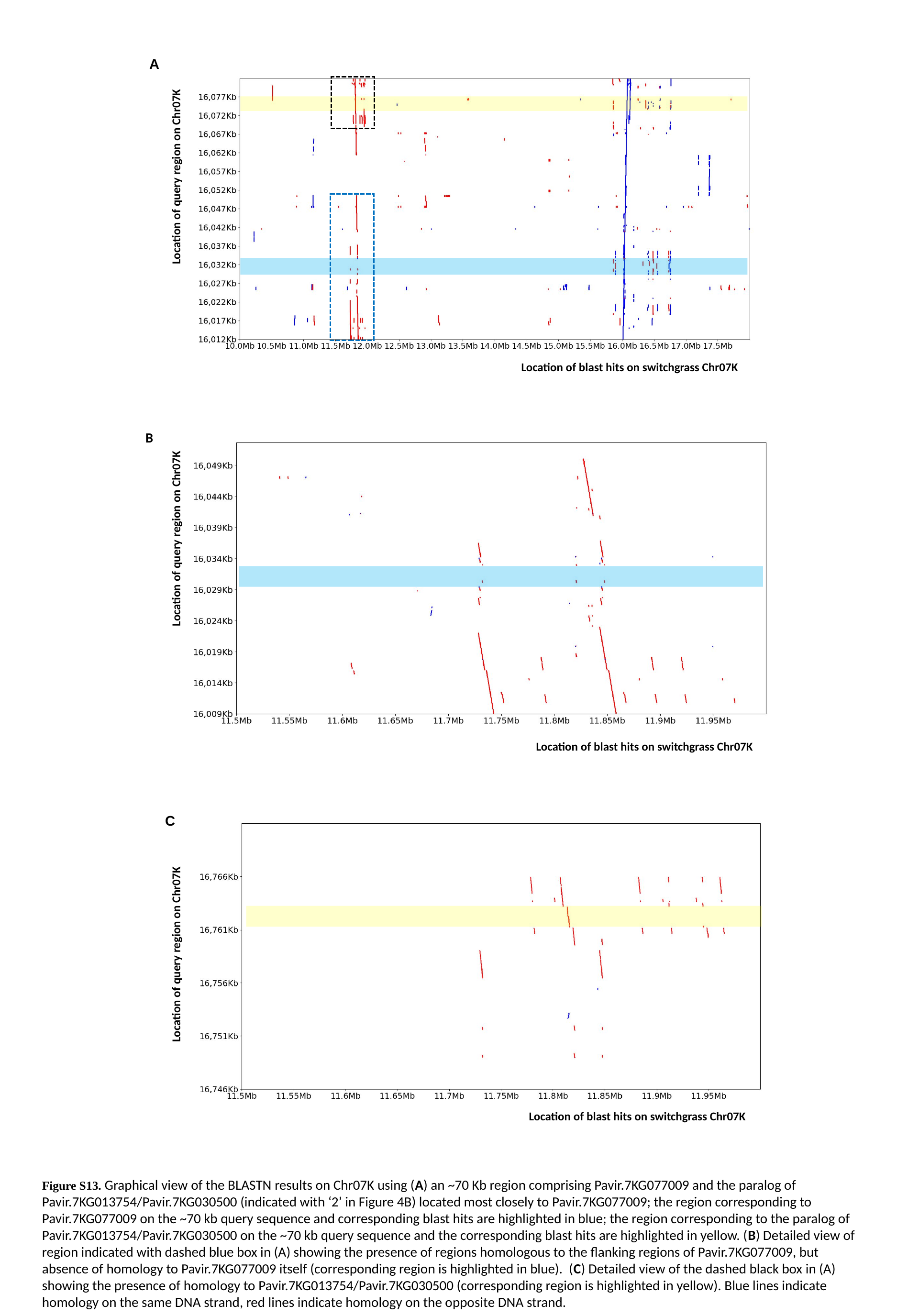

Location of blast hits on switchgrass Chr07K
A
Location of query region on Chr07K
B
Location of query region on Chr07K
Location of blast hits on switchgrass Chr07K
Location of query region on Chr07K
Location of blast hits on switchgrass Chr07K
C
Figure S13. Graphical view of the BLASTN results on Chr07K using (A) an ~70 Kb region comprising Pavir.7KG077009 and the paralog of Pavir.7KG013754/Pavir.7KG030500 (indicated with ‘2’ in Figure 4B) located most closely to Pavir.7KG077009; the region corresponding to Pavir.7KG077009 on the ~70 kb query sequence and corresponding blast hits are highlighted in blue; the region corresponding to the paralog of Pavir.7KG013754/Pavir.7KG030500 on the ~70 kb query sequence and the corresponding blast hits are highlighted in yellow. (B) Detailed view of region indicated with dashed blue box in (A) showing the presence of regions homologous to the flanking regions of Pavir.7KG077009, but absence of homology to Pavir.7KG077009 itself (corresponding region is highlighted in blue). (C) Detailed view of the dashed black box in (A) showing the presence of homology to Pavir.7KG013754/Pavir.7KG030500 (corresponding region is highlighted in yellow). Blue lines indicate homology on the same DNA strand, red lines indicate homology on the opposite DNA strand.
